# Stark choices: exploring health sector costs of policy responses to COVID-19 in low-income and middle-income countries

**DOI:** 10.1136/bmjgh-2021-005759

**Published:** 2021-12-02

**Authors:** Sergio Torres-Rueda, Sedona Sweeney, Fiammetta Bozzani, Nichola R Naylor, Tim Baker, Carl Pearson, Rosalind Eggo, Simon R Procter, Nicholas Davies, Matthew Quaife, Nichola Kitson, Marcus R Keogh-Brown, Henning Tarp Jensen, Nuru Saadi, Mishal Khan, Maryam Huda, Angela Kairu, Raza Zaidi, Edwine Barasa, Mark Jit, Anna Vassall

**Affiliations:** 1 Department of Global Health and Development, London School of Hygiene & Tropical Medicine, London, UK; 2 Department of Infectious Disease Epidemiology, London School of Hygiene & Tropical Medicine, London, UK; 3 Department of Clinical Research, London School of Hygiene & Tropical Medicine, London, UK; 4 Department of Emergency Medicine, Muhimbili University of Health and Allied Sciences, Dar es Salaam, Tanzania; 5 Ifakara Health Institute, Dar es Salaam, Tanzania; 6 Honorary Faculty, The Aga Khan University, Karachi, Pakistan; 7 Department of Community Health Sciences, The Aga Khan University, Karachi, Pakistan; 8 Health Economics Research Unit (HERU), KEMRI-Wellcome Trust Research Programme, Nairobi, Kenya; 9 Health Planning, System Strengthening and Information Analysis Unit, Pakistan Ministry of National Health Services Regulations and Coordination, Islamabad, Pakistan; 10 Nuffield Department of Medicine, University of Oxford, Oxford, UK

**Keywords:** COVID-19, health economics

## Abstract

**Objectives:**

COVID-19 has altered health sector capacity in low-income and middle-income countries (LMICs). Cost data to inform evidence-based priority setting are urgently needed. Consequently, in this paper, we calculate the full economic health sector costs of COVID-19 clinical management in 79 LMICs under different epidemiological scenarios.

**Methods:**

We used country-specific epidemiological projections from a dynamic transmission model to determine number of cases, hospitalisations and deaths over 1 year under four mitigation scenarios. We defined the health sector response for three base LMICs through guidelines and expert opinion. We calculated costs through local resource use and price data and extrapolated costs across 79 LMICs. Lastly, we compared cost estimates against gross domestic product (GDP) and total annual health expenditure in 76 LMICs.

**Results:**

COVID-19 clinical management costs vary greatly by country, ranging between <0.1%–12% of GDP and 0.4%–223% of total annual health expenditure (excluding out-of-pocket payments). Without mitigation policies, COVID-19 clinical management costs per capita range from US$43.39 to US$75.57; in 22 of 76 LMICs, these costs would surpass total annual health expenditure. In a scenario of stringent social distancing, costs per capita fall to US$1.10–US$1.32.

**Conclusions:**

We present the first dataset of COVID-19 clinical management costs across LMICs. These costs can be used to inform decision-making on priority setting. Our results show that COVID-19 clinical management costs in LMICs are substantial, even in scenarios of moderate social distancing. Low-income countries are particularly vulnerable and some will struggle to cope with almost any epidemiological scenario. The choices facing LMICs are likely to remain stark and emergency financial support will be needed.

**Key questions**

**What is already known?**

- The COVID-19 pandemic has ravaged every corner of the world and resource needs of clinical management are estimated to be high: published global annual estimates range from US$130 billion to US$1 trillion and monthly estimates for low-income and middle-income countries (LMICs) range from US$33 to US$61 billion.
- While useful for budgeting purposes, currently available cost estimates of clinical management largely report incremental financial costs based on normative guidelines which are not adequate for priority setting.

**Key questions**

**What are the new findings?**

- We present the first country-specific set of full economic ‘real-world’ costs of COVID-19 clinical management for 79 LMICs which are urgently needed for cost-effectiveness analyses of vaccines, as well as to inform the redesign of essential services packages and to feed into discussions on the balance between broader macroeconomic costs of mitigation strategies and the costs to the health system.
- Average COVID-19 costs vary greatly by country and scenario of social distancing, making up between <0.1%–12% of gross domestic product and 0.4%–223% of total annual health expenditure (excluding out-of-pocket payments).
- Without mitigation policies, average COVID-19 clinical management costs per capita range from US$43.39 to US$75.57, surpassing total annual health expenditure in 22 out of 76 LMICs; in a scenario of moderate social distancing, these drop to US$38.52—US$58.08 and in one of stringent social distancing to US$1.10–US$1.32.

**What do the new findings imply?**

- Health sector costs of COVID-19 are substantial in LMCIs, even when assuming lower-cost critical care options.
- High levels of social distancing by the general population throughout the year would greatly reduce costs to the health system; as social distancing is relaxed, emergency financial support will be needed.

## Introduction

COVID-19 was declared a public health emergency of international concern by the World Health Organization (WHO) in January 2020.^1^ By the end of January 2021, nearly 100 million SARS-CoV2 confirmed infections and over 2.1 million associated deaths had been reported globally.^2^ All-cause excess mortality data in some settings suggest the true figure could be substantially higher.^3 4^ Although clinical data from early in the pandemic suggested only a minority of cases will experience severe (~15%) or critical (~5%) disease that requires hospitalisation,^5^ the estimated resources needed to implement WHO pandemic response guidelines are substantial, particularly for more resource-constrained health systems in low-income and middle-income countries (LMICs).^6^ Although the COVID-19 disease burden in 2021 and beyond is uncertain, particularly with the advent of highly efficacious vaccines, it is unlikely that the disease will be eradicated entirely, so such costs continue to be important for planning and resource allocation.

A limited number of studies have explored country-specific unit costs and total costs of COVID-19 clinical management in high-income settings^7 8^ and LMICs.^9–11^ There have been two efforts to estimate global financing needs, including LMICs. The first was done for 214 countries and territories and costed clinical management, excluding testing, through a financial costing approach. Under different scenarios, the additional yearly health spending at the global level ranged from US$130–231 billion to US$0.6–1 trillion.^12^ A second study, which calculated incremental costs of a health sector-wide response across 73 LMICs (accounting for 95% of the overall population of LMICs), found total recurring financial costs of between US$33 and US$61 billion per month.^13^ Additionally, there are financial costing tools and catalogues available for countries to budget incremental short-term resource requirements.^14–16^

While the abovementioned global studies are key to mobilising resources for COVID-19, they either present incremental financial costs, exclude broader health systems costs or assume a normative approach to resource use unlikely to be followed in LMICs faced with severe resource constraints. Full economic costs of a broader COVID-19 health system response, including ‘real-world’ plausible estimates of service delivery in LMICs under different mitigation scenarios, are urgently needed to inform the priority-setting process. These costs will be required to understand the cost-effectiveness of novel COVID-19 curative and preventive interventions, including those focused on vaccination, as well as to define the extent to which essential services can be maintained during the pandemic. Further, full economic cost data are needed to inform policy choices seeking to balance the broader macroeconomic costs of mitigation strategies against the costs to the health system from the disease. Lastly, country-specific resource estimates are needed to highlight the gaps between currently available financial resources and those which would be required for adequate care and treatment of COVID-19 in LMICs. Such estimates may further contribute to country-specific resource mobilisation efforts.

This paper presents the first estimates of full economic costs of the COVID-19 response to health systems in LMICs taking a ‘real-world’ approach under different pandemic mitigation scenarios over a 12-month period.

## Methods

We used country-specific epidemiological projections from a dynamic transmission model which estimated total numbers of cases, days of hospitalisations and deaths under different mitigation scenarios. We defined the health sector response for three different LMICs in detail (Ethiopia, Pakistan and South Africa), using a combination of guidelines and expert opinion. We used local resource use and price data from a range of primary and secondary data sources. We then extrapolated costs across LMICs at similar income levels. Lastly, we compared cost estimates against country-specific measures of gross domestic product (GDP) and health expenditure. Greater details on our methods can be found in the online Supplementary Methods Appendix.

10.1136/bmjgh-2021-005759.supp1 Supplementary data

### Epidemic mitigation scenarios

Our estimates of COVID-19 cases for different scenarios come from the CovidM epidemiological model, produced by the Centre for the Mathematical Modelling of Infectious Diseases at the London School of Hygiene & Tropical Medicine, which projects the health impact of COVID-19 for 92 LMICs (https://cmmid.github.io/topics/COVID-19/LMIC-projection-reports.html). For each country, the model produces different projections of the number of clinical cases, number of required days in hospital for severe cases (general ward) and critical cases (intensive care unit (ICU)) and deaths for 57 distinct mitigation scenarios that may occur over a 1-year period.^17^

For the costing, four scenarios were chosen. Scenario 1 represents an unmitigated epidemic. While it is unlikely that an epidemic will be unmitigated, it serves as an epidemiological counterfactual to estimate the full costs of COVID-19. Scenarios 2–4 represent a range of plausible levels of mitigation achieved through different policy options: scenario 2 represents a high level of reduction in contacts among symptomatic people and low levels of reduction in contacts in the general population; scenario 3 represents a high level of reduction in contacts among symptomatic people and the general population and scenario 4 represents a 30-day lockdown followed by low levels of reduction in contacts in the general population for the remainder of the year. The online Supplementary Methods Appendix contains further descriptions of the scenarios (table SM3 in the online Supplementary Methods Appendix) and the numbers of cases, days in hospital and deaths for each country and scenario (table SM4 in the online Supplementary Methods Appendix).

### Defining the COVID-19 health sector response

In line with the WHO guidelines, we defined activities for seven priority areas of health sector response to COVID-19 (including both direct service delivery and broader prevention and management strategies): (a) emergency response mechanisms at the national level; (b) risk communication and community engagement; (c) case finding, contact tracing and management; (d) surveillance; (e) public health measures (hygiene education); (f) screening and diagnosis (using polymerase chain reaction, or 'PCR' tests) and (g) case management.^18^ We estimated unit costs per country for each of these activities.

### Estimating unit costs per activity

To estimate the average unit costs for each activity, we used an ingredients-based costing approach.^19^ Detailed inputs costed in each priority area can be found in table SM6 in the online Supplementary Methods Appendix. We calculated full economic costs from a health system perspective over a 12-month time horizon. We used recent local cost and resource use data from three base countries: Ethiopia (low-income country (LIC)), Pakistan (lower middle-income country (lower-MIC)) and South Africa (upper middle-income country (upper-MIC)). As primary data collection from COVID-19 service delivery points was not feasible, we selected countries where we had recently conducted large-scale costing exercises around either tuberculosis (TB) or general health services. These provided current local data on actual resource use, input prices and health system unit cost data for activities such as outpatient consultations, inpatient bed-days and a range of laboratory tests including PCR tests and contact tracing. In the case of Ethiopia and South Africa, we had recent primary data from TB studies (2017–18).^20–22^ In the case of Pakistan, we worked with the Ministry of National Health Services, Regulation and Coordination in 2019–20 to calculate ingredients-based costs for all essential services as part of the Disease Control Priorities 3 project (DCP3).^23^ Although secondary local cost data were used for Pakistan, all costs were subjected to a review by technical working groups as part of DCP3 that included practitioners at all levels of the health system.

### Adapting resource use assumptions to LMICs

We conducted our costing based on global guidelines. However, we adapted the level of COVID-19-specific resource needs to take into account feasibility in LMIC contexts by a combination of reviewing COVID-19 resource planning tools and budgets and scoping literature searches for primary data on clinical care practices in LMICs. Clinical management resource use estimates were adapted based on informal consultations on low-cost critical care options, including an estimation of oxygen therapy needs (see table SM10 in the online Supplementary Methods Appendix). While we had access to and reviewed local COVID-19 data on length of stay in hospital from different settings, this revealed either exceptionally long (early cases) or short (during surge) lengths of stay, and therefore we used data from China and the UK,^24^ in line with the data used in the underlying epidemiological model.

### Extrapolating unit costs from base countries to other LMICs

To generate costs for other LMICs, we extrapolated our detailed unit cost estimates for Ethiopia, Pakistan and South Africa to LICs, lower-MICs and upper-MICs, respectively, based on country-specific epidemiological and health systems data and standard approaches to adjusting prices. In effect, the one constant element between countries is the model of care, with all other aspects of costs adjusted using national-level data in each of the 79 countries.

Each cost input in the ingredients costing was classified as a tradeable good, non-tradeable good or staff cost.^25^ Tradeable goods are generally defined as those that can easily be traded in the international market and include goods such as medical or other supplies and medications. To convert costs of tradeable goods from the base country (eg, Ethiopia) to a ‘second’ country (eg, Afghanistan), we first converted the prices from local currency to 2019 US$ and then apportioned the percentage of the unit cost that was composed of tradeable goods in 2019 US$ from the base country to the second country.

Non-tradeable goods cannot be easily traded in international markets and generally need to be consumed in the country where they have been produced (eg, buildings and utilities). To convert these, we multiplied the proportion of the unit cost that was defined as non-tradeable (in 2019 US$) by the ratio between the 2019 GDP per capita (adjusted for purchasing power parity, or 'PPP') of the second country and the 2019 GDP per capita (adjusted for PPP) of the base country. Data on GDP per capita (adjusted for PPP) were found in the World Bank database.^26^

To convert staff costs from a base country to a second country, we used conversion rates from a regression analysis on wages of health workers for 193 countries to predict wages by country income category relative to GDP per capita. We estimated the number of working hours for nurses, doctors and other medical staff and applied GDP per capita multipliers in order to value their time.^27^

We calculated unit costs per activity for a total of 129 LMICs, as well as a mean unit cost per activity per country income category (LICs, lower-MICs and upper-MICs) weighted by population.

### Calculating total costs

Unit costs per activity were multiplied by the number of activities expected in each country, in some cases driven by the epidemiological estimates (eg, per days in hospital for critical cases) and in others by fixed time and geographical area (eg, per country per day). Since scenario 1 models an unmitigated epidemic only clinical management costs were included. See table SM12 in the online Supplementary Methods Appendix on the number of units used for each activity.

While an effort was made to ensure that the resource use costed is feasible in LMICs, our total cost estimates assume that every patient with severe or critical disease will be hospitalised regardless of existing hospital bed capacity. In other words, we estimate total resource needs regardless of current country-specific non-financial constraints.

Total country-level costs were estimated for 79 LMICs where we had epidemiological estimates. The 79 countries have a combined population of more than 3.98 billion people, which accounts for 60% of the total population of LMICs.^26^ Some LMICs were excluded from our analysis due to the lack of epidemiological estimates or suitable data on GDP with which to make price adjustments.

### Comparing costs

Estimates of annual cost per capita of each scenario in each country were estimated by dividing total costs by the population of each country. These were compared against: (1) country-specific GDP per capita, (2) national health spending (excluding out-of-pocket, or 'OOP', expenditure) per capita, (3) national health spending (including OOP expenditure) per capita and (4) government health expenditure per capita in 76 LMICs where relevant data were available,^26 28^ (see table SM14 in the online Supplementary Methods Appendix). We also present a mean cost per capita per country income category weighted by population.

### Sensitivity analysis

Data on the percentage of symptomatic cases tested with PCR were unavailable and so our assumed base case estimate (10%) was considered highly uncertain. Consequently, we performed a deterministic sensitivity analysis by increasing this value between 20% and 100%.

### Patient and public statement

While there was no patient involvement in our research, we consulted several actors involved in policy-making in LMICs to ensure our work was useful in national-level decision-making.

## Results

### Unit costs in base countries

Table 1 shows the unit costs per activity in our three base countries. Daily case management costs ranged from US$33.32 (Pakistan) to US$105.88 (South Africa) for severe cases and from US$221.18 (Pakistan) to US$1081.94 (South Africa) for critical cases. Costs per case treated ranged from US$266.59 (Pakistan) to US$847.03 (South Africa) for severe cases and from US$2211.83 (Pakistan) to US$10 819.42 (South Africa) for critical cases, assuming 8 days of hospitalisation for severe cases and 10 days for critical cases.^24^ The costs for screening and diagnosis (using PCR) ranged from US$26.98 (Pakistan) to $73.12 (South Africa) per person tested. Unit costs were highest across all activities in South Africa (upper-MIC base country). They were lowest in Pakistan (lower-MIC base country) for activities whose inputs are largely composed of clinical staff time and in Ethiopia (LIC base country) for activities requiring limited or no clinician involvement. The ratios between the highest and lowest unit costs were greatest for non-clinical activities.

**Table 1:** Unit costs per activity for (a) base countries and (b) country income category (population-weighted mean) (2019 US$)

Activity	Unit type	(a) Base countries			(b) Country income category (population-weighted mean)		
		Ethiopia	Pakistan	South Africa	Low-income countries	Lower-middle-income countries	Upper-middle-income countries
1.a. Emergency response mechanisms: National level	Per country per day	$559.26	$778.90	$7697.16	$1197.74	$2697.57	$6317.51
1.b. Emergency response mechanisms: Training of health staff	One-off per site	$4813.58	$8096.53	$68 141.36	$8231.26	$17 600.99	$44 990.67
2. Risk communication and community engagement	Per country per day	$74.14	$91.67	$1133.44	$105.87	$240.57	$558.54
3.a. Case finding, contact tracing and management: Contact tracing	Per person contacted	$3.48	$2.54	$26.23	$3.07	$9.84	$19.68
3.b. Case finding, contact tracing and management: Quarantine of contacts	Per person quarantined	$1.72	$2.35	$29.22	$2.95	$6.36	$16.02
4.a. Surveillance: Case notification	Per positive case	$1.72	$2.35	$29.22	$2.95	$6.36	$16.02
4.b. Surveillance: Reporting (national level)	Per country per week	$3.69	$6.52	$68.26	$7.30	$14.89	$41.07
5. Public health measures: Hygiene education	Per education campaign per month	$44.58	$54.66	$682.05	$63.97	$145.81	$338.43
6. Screening and diagnosis	Per person screened and tested	$36.97	$26.98	$73.12	$31.35	$37.86	$65.30
7.a. Case management: Home-based care	Per person requiring home-based care	$22.90	$12.45	$146.57	$18.55	$51.53	$210.14
7.b. Case management: Hospital based (severe case)	Per day of hospitalisation (severe case)	$35.29	$33.32	$105.88	$35.37	$42.68	$140.53
7.c. Case management: Hospital based (critical case)	Per day of hospitalisation (critical case)	$505.56	$221.18	$1081.94	$310.67	$329.75	$1417.30
7.d. Case management: Death	Per COVID-19-related death	$64.52	$64.52	$64.52	$64.52	$64.52	$64.52

### Extrapolated global unit costs

Table 1 also shows our estimates of the mean unit costs per activity by country income category weighted by population size. Across all activities, unit costs are highest in the upper-MIC category and lowest in the LIC category, except in costs per death where we assume the same costs across all countries. Daily costs for management of severe cases and critical cases ranged from US$35.37 to US$140.53 and from US$310.67 to US$1417.30, respectively. Costs per case treated ranged from US$282.91 to US$1124.24 for severe cases and from US$3106.70 to US$14 172 for critical cases, assuming 8 days of hospitalisation for severe cases and 10 days for critical cases.^24^ The cost person tested with PCR ranged from US$31.35 to US$63.30.

Country-specific unit costs can be found in table SR1 in the online Supplementary Results Appendix. Malaysia had the highest unit costs for the hospital-based case management activities (US$206.38 per day in hospital for severe cases and US$2011.43 for per day in hospital for critical cases) and for testing (US$86.58), while Burundi had the lowest across all three unit costs (US$28.43, US$189.56 and US$25.93, respectively).

10.1136/bmjgh-2021-005759.supp2 Supplementary data

### Total costs and costs per capita

Tables SR2 and SR3 in the online Supplementary Results Appendix show the total costs per country and cost per capita per country, by scenario. Across all scenarios, the total costs per country are highest in India (US$2.10 billion–US$113.70 billion) and lowest in in Sao Tome and Principe (US$863.111–US$10.04 million). It is important to note that the simulation time horizon is 12 months and the epidemic may continue beyond that point so total costs of managing the epidemic in the long term will most likely be higher.

Mean costs per capita per country income group weighted by population are presented in table 2. Costs per capita were similar between scenario 1 (no mitigation) and scenario 4 (30-day lockdown followed by low contact reduction in the general population): between US$43.19–US$75.57 and US$45.73–US$71.62, respectively. Highest costs per capita were observed in scenario 1 in upper-MICs and in scenario 4 for LICs and lower-MICs. Scenario 3 (high levels of contact reduction in symptomatic people and general population) had the lowest costs per capita across all income groups (US$1.10–US$1.32).

**Table 2:** Mean cost per capita by country income category weighted by population (2019 US$)

Scenario	Low-income countries	Lower-middle-income countries	Upper-middle-income countries
Scenario 1: No mitigation	$43.19	$52.63	$75.57
Scenario 2: Contact reduction: high symptomatic cases/low general population	$38.52	$45.96	$58.08
Scenario 3: Contact reduction: high symptomatic cases/high general population	$1.32	$1.10	$1.29
Scenario 4: 30-day lockdown+low contact reduction general population	$45.73	$54.98	$71.62

In all scenarios, the largest cost drivers were screening and diagnosis and case management. Costs of screening and diagnosis were particularly substantial for LICs, accounting for 51.62%–59.47% of total costs, and less substantial for upper-MICs, accounting for 20.10%–26.45% of total costs. Conversely, the costs of case management were particularly substantial for upper-MICs (62.03%–79.90% of total costs), and less so for LICs (37.75%–48.38%). Most of the costs of case management are related to hospital-based critical care (>76% across country income groups and scenarios). Case finding, contact tracing and surveillance and public health measures in contrast made up less than 4% of the total response costs (in scenarios 2–4), see table 3.

**Table 3:** Average percent of total costs by activity, by country income category and by scenario

Activity	Scenario 1: No mitigation			Scenario 2: Contact reduction: high symptomatic cases/low general population			Scenario 3: Contact reduction: high symptomatic cases/high general population			Scenario 4: 30-day lockdown+low contact reduction general population		
	**LIC**	**Lower-MIC**	**Upper-MIC**	**LIC**	**Lower-MIC**	**Upper-MIC**	**LIC**	**Lower-MIC**	**Upper-MIC**	**LIC**	**Lower-MIC**	**Upper-MIC**
1.a. Emergency response mechanisms: National level	0.00%	0.00%	0.00%	0.04%	0.01%	0.02%	1.04%	0.62%	0.99%	0.03%	0.01%	0.02%
1.b. Emergency response mechanisms: Training of health staff	0.00%	0.00%	0.00%	0.08%	0.16%	0.26%	2.19%	6.65%	11.77%	0.06%	0.13%	0.21%
2. Risk communication and community engagement	0.00%	0.00%	0.00%	0.00%	0.00%	0.00%	0.13%	0.08%	0.12%	0.00%	0.00%	0.00%
3.a. Case finding, contact tracing and management: Contact tracing	0.00%	0.00%	0.00%	0.98%	2.27%	1.29%	0.91%	2.00%	1.09%	0.96%	2.22%	1.24%
3.b. Case finding, contact tracing and management: Quarantine of contacts	0.00%	0.00%	0.00%	0.94%	1.21%	1.07%	0.91%	1.11%	0.91%	0.93%	1.18%	1.03%
4.a. Surveillance: Case notification	0.00%	0.00%	0.00%	0.13%	0.17%	0.15%	0.13%	0.16%	0.13%	0.13%	0.17%	0.15%
4.b. Surveillance: Reporting (national level)	0.00%	0.00%	0.00%	0.00%	0.01%	0.01%	0.10%	0.28%	0.56%	0.00%	0.01%	0.01%
5. Public health measures: Hygiene education	0.00%	0.00%	0.00%	0.00%	0.00%	0.00%	0.00%	0.00%	0.00%	0.00%	0.00%	0.00%
6. Screening and diagnosis	51.62%	42.40%	20.10%	59.47%	50.07%	26.45%	56.84%	44.98%	22.39%	58.54%	48.91%	25.36%
7.a. Case management: Home-based care	1.46%	2.85%	2.86%	1.23%	2.45%	2.75%	1.14%	2.15%	2.32%	1.21%	2.39%	2.63%
7.b. Case management: Hospital based (severe case)	8.07%	10.21%	11.86%	6.39%	8.14%	10.47%	6.62%	7.88%	9.19%	6.55%	8.39%	10.68%
7.c. Case management: Hospital based (critical case)	38.37%	44.03%	64.98%	30.36%	35.10%	57.34%	29.59%	33.69%	50.36%	31.18%	36.16%	58.49%
7.d. Case management: Death	0.49%	0.52%	0.20%	0.39%	0.41%	0.18%	0.40%	0.40%	0.15%	0.40%	0.42%	0.18%

LIC, low-income country; Lower-MIC, lower-middle-income country; Upper-MIC, upper-middle-income country.

### Costs as percentage of economic metrics

**Figure 1:**
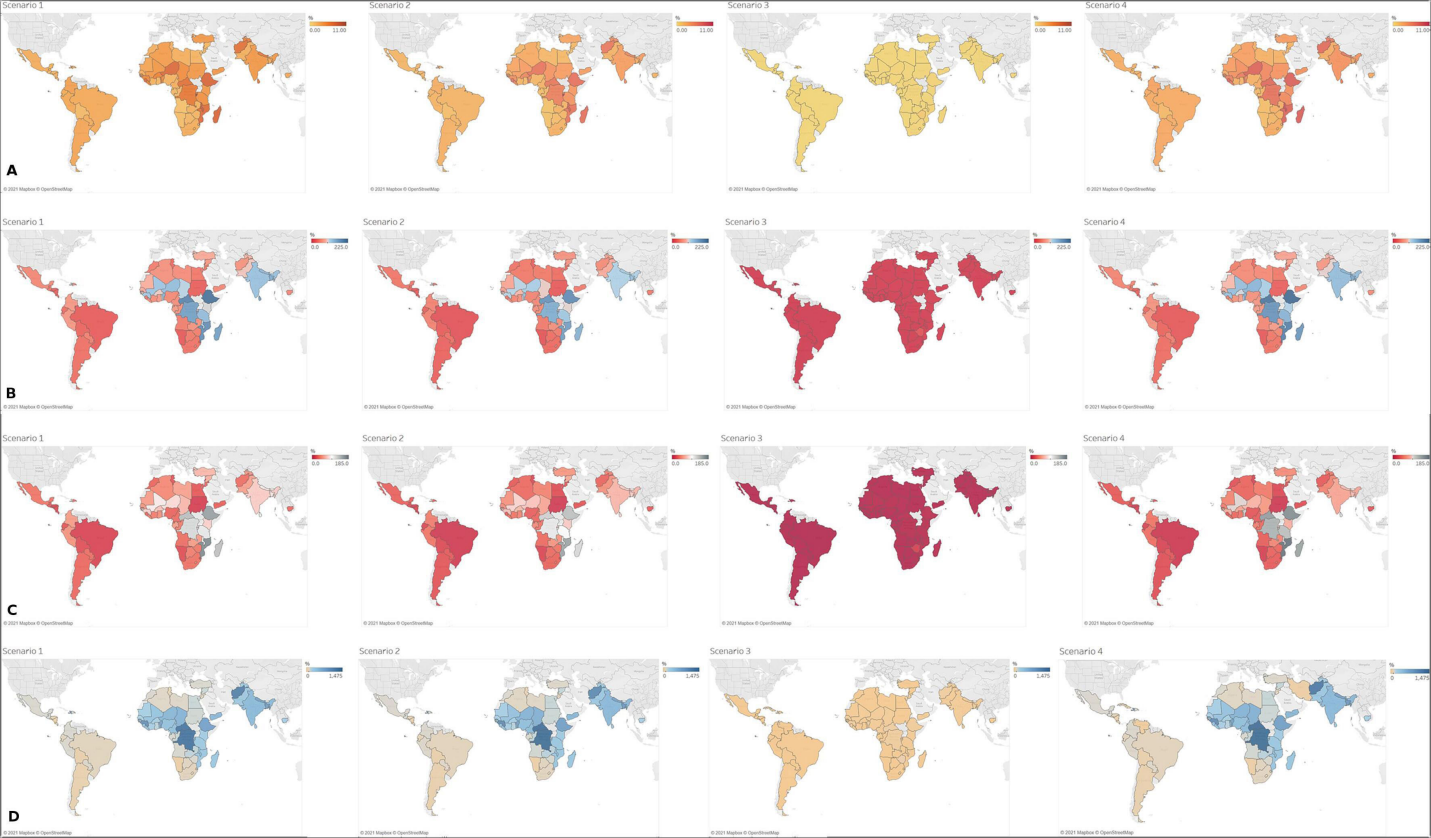
(A): Health System Costs of COVID-19 Response per Capita as % of GDP per Capita (Nominal): Scenario 1: No mitigation; Scenario 2: Contact reduction: high symptomatic cases/low general population; Scenario 3: Contact reduction: high symptomatic cases/high general population; Scenario 4: 30-day lockdown + low contact reduction general population. (B): Health System Costs of COVID-19 Response per Capita as % of Total Health Spending (excl. OOP) per Capita. Scenario 1: No mitigation; Scenario 2: Contact reduction: high symptomatic cases/low general population; Scenario 3: Contact reduction: high symptomatic cases/high general population; Scenario 4: 30-day lockdown + low contact reduction general population. (C): Health System Costs of COVID-19 Response per Capita as % of Total Health Spending (incl. OOP) per Capita. Scenario 1: No mitigation; Scenario 2: Contact reduction: high symptomatic cases/low general population; Scenario 3: Contact reduction: high symptomatic cases/high general population; Scenario 4: 30-day lockdown + low contact reduction general population. (D): Health System Costs of COVID-19 Response per Capita as % of Government Health Spending per Capita. Scenario 1: No mitigation; Scenario 2: Contact reduction: high symptomatic cases/low general population; Scenario 3: Contact reduction: high symptomatic cases/high general population; Scenario 4: 30-day lockdown + low contact reduction general population.

The maps in figure 1A–D (and underlying data in tables SR4-SR7 in the online Supplementary Results Appendix) illustrate and compare the costs per capita of COVID-19 management as a percentage of GDP per capita and of total health spending per capita, using different metrics of health expenditure.

COVID-19 costs per capita as a percentage of GDP per capita are highest in scenario 1 (unmitigated epidemic) and scenario 4 (30-day lockdown and low contact reduction in the general population) across all countries, ranging from 1.43% in Eswatini to 11.85% in Burundi (both scenario 4). They were consistently lowest in scenario 3 (high levels of contact reduction in symptomatic people and general population): between 0.03% in Angola, Bolivia and Ghana and 0.83% in Zimbabwe.

Likewise, COVID-19 costs as a percentage of health expenditure were highest in all countries in scenarios 1 and 4: 23.35%–216.36% and 23.42%–222.34% of total national health spending excluding OOP payments, respectively; 14.68%–171.29% and 15.68%–183.51% of total national health spending including OOP payments, respectively, and 35.15%–1344.28% and 38.24%–1451.34% of government health spending, respectively. Lowest proportions were observed in scenario 3: 0.38%–18.96% of total national health spending excluding OOP payments, 0.26%–15.64% of total national health spending including OOP payments and 0.68%–40.78% of government health spending.

### Sensitivity analysis

We found that estimates were highly sensitive to our assumptions on the number of symptomatic cases tested for scenarios 2–4 (see table SR8 in the online Supplementary Results Appendix). Increasing the number of symptomatic cases tested from 10% to 20% increased our cost per capita estimates by 8%–18%. Assuming that all symptomatic cases would be tested increased our cost per capita estimates by 73%–164%. A sensitivity analysis was not deemed necessary for the unmitigated scenario (scenario 1) as only tests carried out in hospital-based cases were included in the base case.

## Discussion

We provide the first set of country-specific full economic cost estimates of COVID-19 management in LMICs, from a health sector perspective. The countries included in our study account for 60% of the total population of LMICs.^26^ This information can assist policy-makers to better understand trade-offs across all health sector resources and offers an estimate of the scale of financial resources that would be needed for clinical management. Our data may be used for cost-effectiveness analyses of future treatment and prevention strategies, notably vaccines, and to weigh the broader macroeconomic costs of mitigation strategies against the costs to the health system. Additionally, we provide country-specific unit cost estimates for specific COVID-19-related activities that could be useful for planning purposes.

We find that the costs to the health sector of responding to COVID-19 are substantial in LMICs, even when assuming lower-cost critical care options. High levels of social distancing by the general population throughout the year (scenario 3) would greatly reduce costs compared with a policy of allowing the pandemic to proceed unmitigated (scenario 1), but also, importantly, compared with scenarios leading to moderate levels of social distancing (scenarios 2 and 4).

Our findings suggest that the total number of cases is highest in a policy scenario of no mitigation (scenario 1). However, the highest total costs vary by country between scenario 1 and scenario 4 (30-day lockdown followed by minor reductions in contacts). While scenario 4 has a lower number of total cases, costs are comparable to those in scenario 1 because scenario 1 only accounts for clinical management costs and excludes the costs of any mitigation strategies, including testing beyond severe and critical cases in hospital. These results should not be taken as an endorsement that it is preferable, from a financial perspective, to have no mitigation strategy over a strategy of limited social distancing. A no mitigation strategy may result in slightly lower costs of COVID-19 management in some settings but would have substantial knock-on effects on costs and outcomes for other health interventions not quantified in our study.

We compared total COVID-19 costs per country across the four epidemiological scenarios against GDP per capita and three metrics of national-level health expenditure per capita: total national health expenditure with and without OOP payments and government health expenditure. We found that while some countries are likely able to absorb the costs (particularly in scenario 3), even moderate levels of social distancing would lead to high levels of the health spending being directed towards COVID-19 in nearly all countries. In scenario 2, for example, 74 out of 76 countries would need to direct more than 20% of their total national health expenditure (excluding OOP costs) to COVID-19.

COVID-19-related costs would exceed total health spending in several countries, although this varies by expenditure metric examined and epidemiological scenario. COVID-19 costs could exceed total expenditure in between 8 and 11 countries out of 76 when compared against total national health expenditure including OOP. This figure increases to 18–23 countries when compared against total national health expenditure excluding OOP and to 52–54 countries when compared against government health expenditure only. This highlights that in many countries, OOP expenditure and non-government sources of health expenditure, such as donor funding, may play a critical role in covering the costs of the COVID-19 pandemic. As the global macroeconomic situation deteriorates due to the pandemic and overseas development aid is reduced, international agencies and donor nations need to be aware of the potentially catastrophic consequences of reducing funding towards health services in LMICs and the consequent impact on OOP expenditure.

No country is expected to exceed total health expenditure under scenario 3. However, in eight countries, COVID-19 costs are projected to exceed spending across all three health expenditures metrics for scenarios 1, 2 and 4: Burundi, Central African Republic, Democratic Republic of Congo, Ethiopia, The Gambia, Madagascar, Mozambique and Tanzania. These countries, all LICs located in sub-Saharan Africa, are therefore particularly vulnerable and will likely require considerable financial support.

Further research is required to better understand the effect that shocks of this magnitude have on the health system and, particularly, on essential services. It is crucial to understand which services are most vulnerable to being displaced and the levels of funding required to ensure their continued provision, as well as identifying which non-urgent services can be temporarily delayed without causing lasting impact.^29^ While additional funding may aid in ensuring the continuation of some of these services, it may not be possible to relax some of the required infrastructural and human resource constraints in the short term, so the capacity of the health system to absorb additional funding should also be examined.

We carried out a costing from the perspective of the health sector focusing exclusively on COVID-19. We have not quantified the health impacts or costs of deferring other key health sector activities. Further, while high levels of social distancing would lead to better COVID-19-related health outcomes and lower health sector costs, they may also imply economic losses in other sectors as well as social and other non-economic consequences. Decision-makers should consider all these factors when debating COVID-19 mitigation policies.

Our unit cost estimates per day of hospitalisation for severe and critical cases are broadly in line with those published in the literature for LMICs.^9–11^ Our total LMIC costs are lower than those published by Tan-Torres Edejer *et al*,^13^ although they are not entirely comparable due to the sizes of the population studied, the scope of the costing activities included and some key assumptions on resource use, particularly around staff costs. Tan-Torres Edejer *et al* included a larger population in their analysis (countries accounting for 95% of the total population of LMICs, as opposed to 60% in our paper). While the scope of our costing was narrow and focused largely on clinical activities, Tan-Torres Edejer *et al* also included non-clinical interventions (eg, surveillance at points of entry in the country). Lastly, Tan-Torres Edejer *et al* assumed higher remuneration of staff during the pandemic by including hazard pay as per international guidelines, whereas we assumed staff salaries would remain constant with prepandemic salaries.

The two activities with highest proportion of costs in our analysis were screening and diagnosis and case management. Screening and diagnosis costs accounted for a particularly high percentage of total costs in LICs (over 50% of in some scenarios). We assumed all testing across all settings would be PCR based, which led to relatively high unit costs. As the pandemic evolves, we expect less resource intensive diagnostic technologies with adequate accuracy to replace PCR, leading to lower overall costs. Our total costs were highly sensitive to variations in testing scale-up: a 10% increase in the proportion of symptomatic cases tested led to increases of up to 18% in total costs. Country-specific data on numbers of people tested are needed to better calibrate our cost model.

Case management costs were high across settings, particularly in upper-MICs. Healthcare staff salaries made up a large proportion of the costs per day in hospital, particularly for critical cases; higher unit costs in upper-MICs are a product of comparatively higher staff salaries in these settings. Our cost model already assumes a conservative staff-to-patient ratio. We do not expect costs per day of hospitalisation to drop considerably unless this ratio is further reduced. With the advancement of new therapeutics, costs per day of hospitalisation may increase; however, the costs per case treated may decrease if new therapeutics allow for faster patient recovery and reduce length of hospital stay required.

We aimed to calculate overall resource needs, so our total costs assume that all severe and critical patients will be hospitalised. However, this is currently unlikely in certain settings, particularly in LICs, as not all those who need care will be able to access it; ICU capacity remains extremely limited in many settings.^30^ Our costs should therefore not be interpreted as forecasting expenditure, but rather indicative of the scale of financial resources required to provide adequate care at scale.

We did not account for country-specific short-run health system constraints. Accurately quantifying resources constraints, particularly those related to critical care at a global level, is difficult as many components are required (eg, mechanical ventilators, anaesthesiologists, sufficient high-flow oxygen capacity and high clinician-to-patient ratios). It is important for policy-makers to measure and consider these constraints in a country-specific manner when making allocative decisions between different health needs, acknowledging that some resources (eg, human resources) cannot normally be relaxed at scale in the short term, even with additional funding.

While we did not factor access to care in our calculations, we did estimate resource use levels that were considered feasible in LMICs: we assumed, for example, that only one-third of critical cases would receive mechanical ventilation and the other two-thirds would receive other methods of oxygen supplementation. However, such respiratory support is complex and many LMICs may struggle to provide it even in lower quantities. What constitutes a ‘feasible strategies of service delivery’ will inevitably vary between LMICs, but our estimates suggest that, in many settings, even service delivery that is comparatively less resource intensive would lead to very high health sector costs.

Other essential (and less costly) critical care options may be more realistic in certain settings.^31 32^ However, there is scant evidence at present on the effect of different types of critical care pathways, particularly low-cost critical care options, on COVID-19-related mortality. Future work should explore the relationship between costs and outcomes in a more dynamic fashion.

### Limitations

Our methods are subject to several important limitations. First, we rely on epidemiological projections modelled at the start of the epidemic. However, recent data from several settings, notably sub-Saharan Africa, suggest lower numbers of cases and fewer deaths than projected by models (see table SM15 in the online Supplementary Methods Appendix).^2^ The extent to which differences are due to underascertainment of real cases and deaths or to actual differences in epidemic dynamics is unclear. If the former is correct, we would expect numbers of true cases and deaths across LMICs to fall between scenarios 1 and 4 and scenario 3. However, if the latter is correct, our total costs would be largely overestimated. It is important to note, however, that a direct comparison between our modelled estimates and total cases reported to end January 2021 (1 year after the WHO declared COVID-19 a public health emergency of international concern) could also be misleading in some settings. Our modelled data cover a 12-month period per country from the start of the epidemic in each country. However, the epidemic started and accelerated at different times in different countries, so the time horizons considered will differ.

Further, the case numbers that our estimates rely on cover a range of possible epidemic dynamics; one given scenario is unlikely to match the real case numbers currently being observed across all LMICs. Differences between reported and modelled estimates may also be explained by the fact that mitigation policies in place through the pandemic have varied over time in most countries,^33^ and that they may have neither led to comparable levels of contact reduction as in our model nor been implemented for the same amount of time as in our model (1 year from the start of the epidemic).

Second, ‘real-world’ costs are ideally estimated by collecting extensive primary cost data on actual service delivery. We have not been able to do this for COVID-19 and therefore relied on data collected for other purposes and on expert opinion from LMICs to make key assumptions on how services may be delivered. We only used three countries estimates to extrapolate to other settings.

The length of hospital stays necessary for severe and critical cases used in the epidemiological models were based on evidence from early in the pandemic. As more data become available on length of stay and treatment options improve, epidemiological and costing models should be revisited. Service uptake and health-seeking behaviour may also differ by setting and should be considered in further work.

Third, our work focuses on a narrow set of health sector COVID-19 interventions. We do not include the costs of protecting healthcare workers delivering other essential services outside the COVID-19 response (ie, PPE for routine activities) or COVID-19-related costs beyond the health sector (eg, police enforcing social distancing policies), which may be considerable but were beyond the scope of our analysis.

Despite these limitations, our work provides several critical qualitative recommendations for those working in COVID-19 policy-making. First, it is imperative that global agencies and funders continue to ensure sufficient targeted resources are available for LMICs to respond as the pandemic evolves, with most LMICs expected to shift substantial amounts of funding to COVID-19, even with policies of moderate social distancing in place. While much of the focus is on the macroeconomic impact and mortality impact of COVID-19, the fiscal impact on the health sector is likely to be substantial. LICs are particularly vulnerable and some will struggle to cope with almost any COVID-19 scenario. When thinking through mitigation strategies, decision-makers should consider the macroeconomic implications alongside associated potential reductions in healthcare-related costs, including patient costs.

Second, in thinking through resource needs, it is important for countries to re-evaluate interventions and adapt response measures in ways that are context appropriate, affordable and sustainable, particularly in relation to high-cost activities, namely screening and testing and hospital-based care. This could include intervention delivery re-design and adaptations such as integration of care, leveraging of community health workers and home-based care, better targeting of interventions such as testing, and lower cost diagnostic approaches and critical care, among other ideas.

Finally, while our results reflect the myriad decisions about care, protection and patient experience that are required to plan resource use, there is little discussion or data on what is feasible in LMICs. This is a task that cannot be met using a global perspective but needs country-specific inputs to reflect the health system characteristics of each country. We therefore also call for urgent support to encourage interaction of economists, planners, service managers and epidemiological modellers to inform COVID-19 policy at the country level across LMICs.

## Conclusion

We present the first dataset of COVID-19 clinical management costs across LMICs. These data can be used for cost-effectiveness analyses of prevention strategies, notably vaccines, and can assist policy-makers understand trade-offs between essential services as well as inform discussions on the balance between broader macroeconomic costs of mitigation strategies and health sector costs. We find that COVID-19 clinical management costs are substantial in LMICs, even in scenarios of moderate social distancing and assuming lower-cost critical care options. LICs are particularly vulnerable and some will struggle to cope with almost any COVID-19 scenario. As social distancing is relaxed, emergency financial support will be needed. The choices facing LMICs are likely to remain stark.

## Data Availability

All data relevant to the study are included in the article or uploaded as supplementary information.
