# Supplementary material for: Stark choices: exploring health sector costs of policy responses to COVID-19 in low-income and middle-income countries
Source: BMJ Glob Health. 2021 Dec 2;6(12):e005759. doi: 10.1136/bmjgh-2021-005759 (PMC8640196; doi:10.1136/bmjgh-2021-005759)
Supplement: Supplementary data [file bmjgh-2021-005759supp001.pdf]

Supplementary Methods Appendix

Table of Contents

1. Epidemiological model 4
  1.1 Parameters used in epidemiological model 4
    1.1.1 Flow diagram showing compartments and flows in the epidemiological model (Figure SM1) 4
    1.1.2 Table SM1: General model parameters 5
    1.1.3 Table SM2: Age-specific hospitalisation and fatality risk 6
  1.2 Scenarios 6
    1.2.1 Table SM3: Scenario descriptions 7
  1.3 Table SM4: Expected number of cases, days of hospitalisations (ICU and non-ICU) and deaths per country per scenario per year 8
2. Health resource use and costing parameters and assumptions 15
  2.1. Summary 15
  2.2 Calculation of unit costs per activity for three base countries 16
    2.2.1 General Approach 16
    2.2.2 Intervention costs 16
    2.2.3 Table SM5: Activities and unit types 17
    2.2.4 Defining inputs, inputs quantities and input costs 17
    2.2.5 Table SM6: Quantities and unit costs per input per activity per country 18
    2.2.6 Input quantities 20
    2.2.7 Input unit costs 20
      2.2.7.1 Estimation of non-bed-day costs (Pakistan) 20
      2.2.7.2 Estimation of non-bed-day costs (Ethiopia and South Africa) 21
      2.2.7.3 Price adjustments 22
      2.2.7.4 Table SM7: Relative GDP adjustment factors 22
      2.2.7.5 Estimation of bed-day costs (all countries) 22
      2.2.7.6 COVID-19 specific costs 23
      2.2.7.7 Table SM8: PPE costs per general ward bed day and per ICU bed day 24
      2.2.7.8 Table SM9: Hygiene costs per general ward and ICU bed day 25
      2.2.7.9 Table SM10: Oxygen supplementation assumptions 26
  2.3 Extrapolation of unit costs in base countries to calculate unit costs across LICs, Lower-MICs and Upper-MICs 27
    2.3.1 Table SM11: Health worker earnings as a multiple of GDP per capita 28
  2.4 Calculation of country-specific number of units per activity 29
    2.4.1 Table SM12: Number of country-specific units per activity 29
    2.4.2. Table SM13: Test positivity rate by country and average 31

2.5 Country-specific per capita costs and per capita costs as a proportion of gross domestic product (GDP) per capita and other measures of health expenditure per capita31
2.5.1 Table SM14: Population, GDP and health spending per country 32
2.6 Confirmed cases to date 36
2.6.1 Table SM15: Number of confirmed cases of COVID-19 up to 31 January 2021 .. 36
3. References 38

1. Epidemiological model

1.1 Parameters used in epidemiological model

1.1.1 Flow diagram showing compartments and flows in the epidemiological model (Figure SM1)

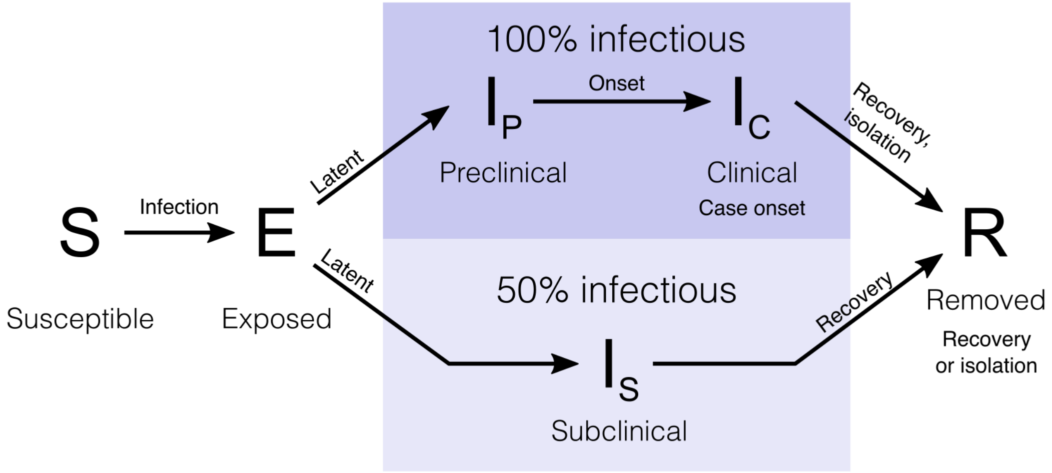

1.1.2 Table SM1: General model parameters

Parameter	Description	Value	Reference
d_E	Latent period (E to I_P and E to I_S; days)	Gamma distribution (μ=4.0, k=4)	(1)
d_P	Duration of preclinical infectiousness (I_P to I_C; days)	Gamma distribution (μ=2.4, k=4)	(1)
d_C	Duration of clinical infectiousness (I_C to R; days)	Gamma distribution (μ=3.2, k=3.7)	(2)
d_S	Duration of subclinical infectiousness (I_S to R; days)	Gamma distribution (μ=7.0, k=4.0)	Assumed
	Incubation period (E to I_C; days)	$d_E + d_P$; mean 6.4 days	Derived
	Serial interval (days)	$d_E + (y_i(d_P + d_C) + (1 - y_i)d_S)/2$; mean approximately 7 days	Derived
u	Susceptibility to infection on contact	Calculated from R_0	Derived
y_i	Probability of clinical symptoms on infection for age group *i*	Estimated from case distributions across 6 countries	(3)
f	Relative infectiousness of subclinical cases	50%	Assumed
c_{ij}	Number of age-*j* individuals contacted by an age-*i* individual per day	Country-specific contact matrix	(4)
N_i	Number of age-*i* individuals	Demographic data	(5)
Δt	Time step for discrete-time simulation	0.25 days	
	Delay from onset to hospitalisation (days)	Gamma distribution (μ=7.0, k=5.0)	
	Duration of hospitalisation in non-ICU bed, severe case (days)	Gamma distribution (μ=14.6, k=5.0)	(6)
	Duration of hospitalisation in non-ICU bed, critical case (before ICU bed; days)	Gamma distribution (μ=6.0, k=5.0)	
	Duration of hospitalisation in ICU bed, critical case (after non-ICU bed; days)	Gamma distribution (μ=9.6, k=5.0)	(7-13)
	Delay from onset to death (days)	Gamma distribution (μ=22, k=10)	(14, 15)

1.1.3 Table SM2: Age-specific hospitalisation and fatality risk

Age group	Case-fatality risk	% of cases hospitalised	% of hospital patients needing ICU
0–9	0.00%	0.0%	30%
1–10	0.09%	0.8%	30%
20-29	0.10%	0.8%	30%
30-39	0.12%	1.0%	30%
40-49	0.23%	1.9%	30%
50-59	0.68%	5.4%	30%
60-69	1.87%	15.1%	30%
70-79	4.14%	33.3%	30%
80+	7.68%	61.8%	30%

Source: Davies et al (2020) (16)

1.2 Scenarios

The epidemiological model uses data from low- and middle-income countries. For each country, the model produces estimates on the number of cases, hospitalisations, number of days in hospital for severe cases (general ward) and critical cases (intensive care unit), and deaths for 57 distinct epidemiological scenarios (2).

For this study, four epidemiological scenarios were chosen out of the set of 57 possible scenarios. Scenario 1 represents an unmitigated epidemic. Scenarios 2-4 scenarios were chosen because they represent a variety of plausible policy options. Descriptions of the scenarios are presented below in Table SM3. Number of cases, days in hospital and deaths per country per scenario can be found in Table SM4.

1.2.1 Table SM3: Scenario descriptions

**Scenario 1**	Unmitigated epidemic: no mitigation policies are introduced, and there are no reductions in contacts across any population or setting.
**Scenario 2**	The whole population is covered in this intervention scenario. The intervention is triggered by daily incidence reaching 1 per 10,000. The intervention includes self-isolation of symptomatic persons for duration of symptoms, modelled as an additional reduction in contacts among symptomatic people of 75 %. The intervention includes distancing measures that reduce contacts at school by 20 %, at work by 20 %, in other settings by 20 %, and in the home setting by 0%. There is no difference in intervention by age.
**Scenario 3**	The whole population is covered in this intervention scenario. The intervention is triggered by daily incidence reaching 1 per 10,000. The intervention includes self-isolation of symptomatic persons for duration of symptoms, modelled as an additional reduction in contacts among symptomatic people of 75 %. The intervention includes distancing measures that reduce contacts at school by 80 %, at work by 80 %, in other settings by 80 % and in the home setting by 0 %. There is no difference in intervention by age.
**Scenario 4**	49.The intervention is temporary lockdown (30 days) which leads to 100 % of the population reducing their contacts through school, home, work and other settings by 100%, 0%, 37.5 % and 37.5 %, respectively, during Lockdown occurs for the first 30 days. After lockdown is lifted 100% of the population reduces their contacts through school, home, work and other settings by 20 %, 0%, 20% and 20%, respectively.

*For reference, these correspond to Scenarios 1, 4, 22 and 49 in the CovidM epidemiological model, respectively.

1.3 Table SM4: Expected number of cases, days of hospitalisations (ICU and non-ICU) and deaths per country per scenario per year

Country	Scenario	Total Number of Cases per Year	Total Number of ICU bed days per Year	Total Number of Non-ICU Bed Days per Year	Total Number of Deaths per Year
Afghanistan	1	12,218,384	1,644,757	3,068,969	102,557
Afghanistan	2	9,128,640	1,124,522	2,096,065	69,980
Afghanistan	3	5,057,917	659,168	1,228,500	40,911
Afghanistan	4	10,687,759	1,379,972	2,571,196	85,767
Angola	1	10,568,602	1,337,286	2,495,053	82,997
Angola	2	7,951,659	958,471	1,790,227	59,579
Angola	3	3,787,000	480,525	904,768	29,918
Angola	4	9,243,636	1,153,109	2,151,984	71,543
Argentina	1	16,773,845	7,129,192	13,331,843	443,779
Argentina	2	12,167,825	4,923,904	9,183,093	306,425
Argentina	3	7,206,744	3,179,287	5,930,435	197,212
Argentina	4	14,559,346	6,161,078	11,508,390	383,318
Burundi	1	3,825,576	487,746	912,821	30,294
Burundi	2	2,875,192	343,199	643,374	21,458
Burundi	3	1,563,194	192,617	359,761	11,941
Burundi	4	3,360,358	416,410	783,449	25,959
Benin	1	3,987,008	610,334	1,140,853	37,967
Benin	2	3,027,031	436,071	813,544	27,099
Benin	3	1,617,554	243,858	455,765	15,181
Benin	4	3,517,493	523,440	980,738	32,601
Burkina Faso	1	6,855,948	954,395	1,781,199	59,385
Burkina Faso	2	5,231,105	692,107	1,290,565	42,965
Burkina Faso	3	2,954,321	421,063	787,441	26,090
Burkina Faso	4	6,035,206	826,512	1,546,413	51,440
Bangladesh	1	57,227,127	14,236,788	26,575,364	885,269
Bangladesh	2	43,270,144	10,038,443	18,753,926	623,995
Bangladesh	3	23,696,469	5,910,527	11,038,187	366,351
Bangladesh	4	50,407,176	12,324,089	23,001,527	766,395
Bolivia	1	3,881,885	929,771	1,734,889	57,656
Bolivia	2	2,855,102	614,416	1,146,287	38,054
Bolivia	3	1,246,487	279,999	519,997	17,368
Bolivia	4	3,364,052	778,136	1,449,733	48,330
Brazil	1	76,815,441	27,418,043	51,183,619	1,705,997
Brazil	2	56,285,085	18,283,102	34,119,544	1,139,192
Brazil	3	30,752,013	10,628,491	19,851,593	658,619
Brazil	4	66,729,482	23,242,139	43,395,705	1,445,675
Botswana	1	765,573	159,461	296,629	9,918
Botswana	2	565,036	107,208	199,542	6,660
Botswana	3	305,082	61,841	114,201	3,789
Botswana	4	662,702	134,418	250,989	8,334
Central African Republic	1	1,542,576	227,256	422,653	14,108
Central African Republic	2	1,168,851	158,952	297,691	9,900
Central African Republic	3	696,607	102,635	192,340	6,435
Central African Republic	4	1,353,904	194,599	363,301	12,130
Cote d'Ivoire	1	8,535,012	1,316,578	2,457,106	82,053
Cote d'Ivoire	2	6,298,092	916,128	1,708,562	57,156

Country	Scenario	Total Number of Cases per Year	Total Number of ICU bed days per Year	Total Number of Non-ICU Bed Days per Year	Total Number of Deaths per Year
Cote d'Ivoire	3	2,781,975	429,891	803,017	26,661
Cote d'Ivoire	4	7,403,109	1,119,330	2,085,969	69,716
Cameroon	1	8,729,175	1,249,335	2,335,084	77,781
Cameroon	2	6,604,562	889,631	1,661,757	55,432
Cameroon	3	3,552,194	503,336	939,580	31,265
Cameroon	4	7,690,770	1,078,673	2,014,896	67,062
Congo, Dem. Rep.	1	28,914,535	4,131,603	7,713,654	257,149
Congo, Dem. Rep.	2	21,729,089	2,925,816	5,459,743	181,725
Congo, Dem. Rep.	3	10,339,605	1,480,493	2,755,736	91,223
Congo, Dem. Rep.	4	25,274,207	3,537,680	6,601,661	219,628
Congo, Rep.	1	1,819,210	284,222	531,531	17,714
Congo, Rep.	2	1,380,686	201,095	376,299	12,564
Congo, Rep.	3	828,849	128,924	241,162	8,085
Congo, Rep.	4	1,603,300	245,240	457,934	15,290
Colombia	1	18,341,207	6,156,014	11,493,004	382,745
Colombia	2	13,426,478	4,130,466	7,696,118	256,668
Colombia	3	7,192,838	2,342,407	4,372,127	144,933
Colombia	4	15,964,753	5,231,315	9,754,859	325,054
Comoros	1	290,557	47,011	87,853	2,912
Comoros	2	215,335	32,916	61,231	2,047
Comoros	3	114,052	18,551	34,354	1,129
Comoros	4	252,887	40,322	75,628	2,512
Cabo Verde	1	181,545	41,379	77,437	2,566
Cabo Verde	2	128,006	26,548	49,137	1,638
Cabo Verde	3	67,261	14,657	27,550	905
Cabo Verde	4	153,456	33,985	63,203	2,102
Costa Rica	1	1,822,573	652,845	1,218,194	40,619
Costa Rica	2	1,326,971	428,627	799,724	26,659
Costa Rica	3	745,696	252,693	470,210	15,641
Costa Rica	4	1,578,679	549,464	1,023,619	34,174
Dominican Republic	1	3,807,777	1,127,809	2,105,557	70,140
Dominican Republic	2	2,808,435	765,172	1,422,812	47,526
Dominican Republic	3	1,562,725	456,253	850,089	28,343
Dominican Republic	4	3,315,475	964,871	1,796,265	59,882
Algeria	1	14,034,753	3,466,815	6,462,518	215,453
Algeria	2	9,901,970	2,171,944	4,050,559	135,157
Algeria	3	5,054,134	1,144,758	2,139,221	70,840
Algeria	4	11,937,307	2,835,058	5,295,542	175,989
Ecuador	1	6,238,254	1,828,487	3,416,055	113,770
Ecuador	2	4,681,266	1,256,087	2,337,136	77,960
Ecuador	3	2,586,022	739,506	1,376,652	45,789
Ecuador	4	5,480,200	1,567,059	2,923,490	97,214
Egypt, Arab Rep.	1	32,689,306	7,149,998	13,352,308	444,818
Egypt, Arab Rep.	2	23,251,250	4,594,336	8,576,020	285,393
Egypt, Arab Rep.	3	11,840,379	2,461,239	4,607,143	152,674
Egypt, Arab Rep.	4	27,975,741	5,946,559	11,123,015	370,198
Ethiopia	1	37,424,355	6,123,830	11,438,760	380,821
Ethiopia	2	28,272,227	4,318,605	8,066,304	268,718

**Country**	**Scenario**	**Total Number of Cases per Year**	**Total Number of ICU bed days per Year**	**Total Number of Non-ICU Bed Days per Year**	**Total Number of Deaths per Year**
Ethiopia	3	14,996,880	2,457,012	4,593,452	152,671
Ethiopia	4	32,804,210	5,277,773	9,861,571	328,540
Gabon	1	752,440	127,913	239,231	7,960
Gabon	2	572,426	89,835	168,146	5,614
Gabon	3	347,308	58,095	109,709	3,636
Gabon	4	664,128	110,378	206,162	6,868
Ghana	1	10,378,959	1,826,469	3,406,632	113,608
Ghana	2	7,713,894	1,273,479	2,379,059	79,390
Ghana	3	3,423,372	602,270	1,120,060	37,244
Ghana	4	9,039,576	1,557,544	2,901,100	96,753
Guinea	1	4,258,486	700,273	1,311,264	43,627
Guinea	2	3,197,000	509,157	950,869	31,664
Guinea	3	1,702,115	304,198	565,934	18,716
Guinea	4	3,718,928	614,331	1,144,140	38,188
Gambia, The	1	787,667	120,450	224,342	7,491
Gambia, The	2	592,871	88,337	163,947	5,443
Gambia, The	3	324,942	53,737	101,076	3,350
Gambia, The	4	686,599	104,927	196,707	6,559
Guinea-Bissau	1	646,616	100,306	186,821	6,208
Guinea-Bissau	2	490,671	72,272	135,491	4,506
Guinea-Bissau	3	284,321	45,434	83,769	2,782
Guinea-Bissau	4	569,960	87,201	163,160	5,406
Equatorial Guinea	1	471,118	62,021	116,074	3,858
Equatorial Guinea	2	359,113	44,812	83,462	2,772
Equatorial Guinea	3	194,874	25,338	47,642	1,594
Equatorial Guinea	4	416,197	53,836	100,579	3,349
Guatemala	1	5,931,816	1,188,378	2,215,139	73,895
Guatemala	2	4,445,955	809,411	1,509,951	50,398
Guatemala	3	2,471,212	477,648	888,300	29,576
Guatemala	4	5,200,461	1,004,989	1,873,663	62,446
Honduras	1	3,368,666	711,832	1,328,439	44,068
Honduras	2	2,554,279	498,195	928,829	30,884
Honduras	3	1,508,915	311,766	579,276	19,368
Honduras	4	2,966,095	611,292	1,145,329	38,069
Haiti	1	3,844,733	864,008	1,611,689	53,684
Haiti	2	2,891,884	602,646	1,127,805	37,539
Haiti	3	1,628,745	363,202	678,225	22,680
Haiti	4	3,378,438	746,954	1,392,145	46,507
India	1	485,771,554	134,139,960	250,304,013	8,338,438
India	2	365,933,322	93,048,830	173,656,182	5,785,607
India	3	217,279,232	58,353,362	108,949,367	3,620,179
India	4	427,295,821	115,025,841	214,808,062	7,154,678
Iraq	1	13,378,016	2,160,706	4,036,577	134,704
Iraq	2	10,055,987	1,513,885	2,832,294	94,397
Iraq	3	5,444,676	850,809	1,586,861	52,768
Iraq	4	11,745,267	1,852,021	3,459,985	115,382

Country	Scenario	Total Number of Cases per Year	Total Number of ICU bed days per Year	Total Number of Non-ICU Bed Days per Year	Total Number of Deaths per Year
Jordan	1	3,230,121	545,182	1,014,537	33,793
Jordan	2	2,403,685	360,587	671,528	22,396
Jordan	3	1,441,002	226,241	421,434	14,020
Jordan	4	2,802,082	455,066	851,418	28,349
Kenya	1	17,932,718	3,334,486	6,220,435	207,231
Kenya	2	13,972,380	2,685,063	5,015,623	166,727
Kenya	3	8,920,887	1,777,161	3,304,638	110,214
Kenya	4	15,940,230	3,027,794	5,654,038	188,428
Cambodia	1	5,249,630	1,075,358	2,009,207	67,003
Cambodia	2	3,735,697	699,366	1,307,952	43,433
Cambodia	3	1,491,662	285,706	532,484	17,650
Cambodia	4	4,455,702	883,330	1,656,498	55,017
Lebanon	1	2,473,142	849,633	1,578,978	52,778
Lebanon	2	1,838,521	596,389	1,111,151	36,992
Lebanon	3	1,166,532	410,943	767,150	25,628
Lebanon	4	2,165,968	736,594	1,374,880	45,888
Liberia	1	1,679,614	288,953	537,755	17,919
Liberia	2	1,277,408	204,826	383,902	12,778
Liberia	3	740,784	127,259	237,192	7,905
Liberia	4	1,478,591	249,999	464,731	15,428
Libya	1	2,278,127	480,466	897,001	29,873
Libya	2	1,632,675	312,686	585,736	19,496
Libya	3	836,827	167,081	312,036	10,330
Libya	4	1,947,757	401,241	748,906	24,938
Sri Lanka	1	8,020,343	3,408,470	6,355,545	211,681
Sri Lanka	2	5,916,153	2,373,553	4,436,367	147,652
Sri Lanka	3	3,594,782	1,558,313	2,903,475	96,594
Sri Lanka	4	7,016,699	2,945,770	5,508,073	183,972
Lesotho	1	696,741	145,159	271,321	9,017
Lesotho	2	508,495	95,194	178,041	5,963
Lesotho	3	229,199	44,272	82,772	2,796
Lesotho	4	600,007	121,391	224,865	7,490
Morocco	1	13,242,617	4,386,424	8,186,000	272,409
Morocco	2	9,819,053	3,059,154	5,703,002	190,014
Morocco	3	5,899,932	1,973,056	3,680,792	122,031
Morocco	4	11,587,125	3,784,741	7,071,203	235,509
Madagascar	1	9,150,387	1,448,350	2,707,203	90,126
Madagascar	2	6,782,766	1,015,312	1,897,326	63,301
Madagascar	3	3,564,207	563,428	1,049,184	35,010
Madagascar	4	7,966,525	1,244,624	2,321,257	77,440
Mexico	1	46,024,529	13,801,740	25,788,653	858,167
Mexico	2	34,417,216	9,459,831	17,652,018	588,526
Mexico	3	21,097,466	6,179,136	11,554,983	384,123
Mexico	4	40,449,265	11,850,319	22,128,972	737,193
Mali	1	6,261,539	750,362	1,399,913	46,770
Mali	2	4,479,077	497,582	928,693	30,937

Country	Scenario	Total Number of Cases per Year	Total Number of ICU bed days per Year	Total Number of Non-ICU Bed Days per Year	Total Number of Deaths per Year
Mali	3	2,225,009	265,779	494,222	16,416
Mali	4	5,359,818	623,714	1,164,621	38,817
Mozambique	1	10,132,076	1,434,436	2,675,564	89,192
Mozambique	2	7,529,591	1,000,297	1,869,917	62,413
Mozambique	3	3,939,631	555,318	1,035,086	34,251
Mozambique	4	8,820,157	1,230,587	2,293,160	76,324
Mauritania	1	1,535,219	254,586	475,933	15,815
Mauritania	2	1,165,276	182,692	339,830	11,333
Mauritania	3	707,750	121,284	227,135	7,524
Mauritania	4	1,353,942	221,412	412,272	13,723
Mauritius	1	486,886	209,370	390,332	13,048
Mauritius	2	351,305	138,424	258,994	8,630
Mauritius	3	214,210	88,073	165,625	5,463
Mauritius	4	422,541	176,489	329,163	11,016
Malawi	1	6,206,021	853,281	1,590,559	52,896
Malawi	2	4,609,106	597,184	1,114,079	37,180
Malawi	3	2,414,872	329,973	616,215	20,406
Malawi	4	5,394,534	731,215	1,362,680	45,338
Namibia	1	805,969	113,125	211,467	7,032
Namibia	2	594,975	78,583	146,713	4,861
Namibia	3	271,680	36,561	68,779	2,297
Namibia	4	696,765	95,834	179,066	5,975
Niger	1	7,479,841	911,995	1,704,345	56,836
Niger	2	5,361,960	604,502	1,131,298	37,571
Niger	3	2,656,778	323,439	603,881	20,052
Niger	4	6,407,713	758,281	1,417,828	47,137
Nigeria	1	67,998,172	10,931,387	20,404,287	679,438
Nigeria	2	51,622,543	7,976,219	14,878,213	495,447
Nigeria	3	28,071,653	4,656,256	8,692,205	289,284
Nigeria	4	59,789,002	9,541,920	17,807,959	593,372
Nicaragua	1	2,235,573	500,811	937,663	31,193
Nicaragua	2	1,671,928	340,381	632,681	21,124
Nicaragua	3	917,568	190,083	355,496	11,913
Nicaragua	4	1,954,965	422,936	790,201	26,308
Nepal	1	9,915,595	2,499,734	4,665,603	155,339
Nepal	2	7,426,283	1,746,579	3,269,303	108,874
Nepal	3	3,596,042	931,578	1,736,856	57,693
Nepal	4	8,680,619	2,156,185	4,028,306	134,341
Pakistan	1	71,833,291	13,106,615	24,490,691	816,437
Pakistan	2	53,017,173	8,893,216	16,571,106	551,824
Pakistan	3	29,435,592	5,229,563	9,784,165	324,960
Pakistan	4	62,541,662	10,974,825	20,499,025	682,425
Peru	1	11,655,014	3,620,110	6,750,234	224,803
Peru	2	8,460,495	2,403,019	4,479,917	148,873
Peru	3	4,169,861	1,246,737	2,324,155	77,272
Peru	4	10,081,294	3,039,428	5,677,002	188,946

Country	Scenario	Total Number of Cases per Year	Total Number of ICU bed days per Year	Total Number of Non-ICU Bed Days per Year	Total Number of Deaths per Year
Paraguay	1	2,401,205	602,572	1,122,938	37,456
Paraguay	2	1,771,743	398,632	744,467	24,878
Paraguay	3	880,974	208,729	389,899	12,897
Paraguay	4	2,085,255	508,237	946,491	31,507
West Bank and Gaza	1	1,594,004	226,571	422,739	14,105
West Bank and Gaza	2	1,189,223	151,666	281,435	9,407
West Bank and Gaza	3	712,803	95,453	176,326	5,949
West Bank and Gaza	4	1,387,925	190,382	355,232	11,799
Rwanda	1	4,260,092	653,279	1,221,704	40,608
Rwanda	2	3,212,135	457,874	855,830	28,478
Rwanda	3	1,749,170	255,902	479,834	16,022
Rwanda	4	3,746,403	557,317	1,041,050	34,717
Sudan	1	14,419,983	2,514,131	4,680,809	155,985
Sudan	2	10,877,483	1,772,406	3,314,864	110,204
Sudan	3	5,777,423	1,008,372	1,878,301	62,797
Sudan	4	12,629,479	2,164,741	4,038,215	134,409
Senegal	1	5,506,930	943,941	1,765,633	58,634
Senegal	2	4,152,656	688,535	1,284,545	42,863
Senegal	3	2,285,377	427,813	800,324	26,503
Senegal	4	4,809,001	828,514	1,548,750	51,441
Sierra Leone	1	2,630,770	424,740	793,710	26,429
Sierra Leone	2	1,994,333	307,273	574,350	19,120
Sierra Leone	3	1,158,003	191,212	360,302	12,061
Sierra Leone	4	2,319,759	370,962	690,723	22,967
El Salvador	1	2,258,282	726,964	1,354,593	45,161
El Salvador	2	1,664,922	490,166	916,504	30,630
El Salvador	3	908,668	289,894	538,905	17,906
El Salvador	4	1,965,853	619,691	1,156,954	38,540
Sao Tome and Principe	1	72,284	11,232	20,945	696
Sao Tome and Principe	2	54,913	8,014	15,110	500
Sao Tome and Principe	3	29,482	4,448	8,484	284
Sao Tome and Principe	4	63,572	9,662	17,971	607
Eswatini	1	363,364	62,144	115,928	3,869
Eswatini	2	266,346	40,924	76,891	2,558
Eswatini	3	119,568	18,886	35,997	1,187
Eswatini	4	313,533	51,988	96,274	3,204
Syrian Arab Republic	1	5,792,480	989,565	1,847,430	61,580
Syrian Arab Republic	2	4,370,152	718,417	1,339,838	44,443
Syrian Arab Republic	3	2,590,708	461,949	861,860	28,637
Syrian Arab Republic	4	5,101,507	858,728	1,601,690	53,349
Chad	1	5,065,943	601,091	1,124,428	37,285
Chad	2	3,620,550	399,236	745,415	24,835
Chad	3	1,795,298	213,053	398,406	13,124
Chad	4	4,330,614	500,213	933,773	31,055
Togo	1	2,738,322	409,696	766,659	25,516
Togo	2	2,084,482	294,395	549,644	18,294

Country	Scenario	Total Number of Cases per Year	Total Number of ICU bed days per Year	Total Number of Non-ICU Bed Days per Year	Total Number of Deaths per Year
Togo	3	1,117,766	165,864	310,016	10,261
Togo	4	2,416,838	353,372	660,994	21,999
Tajikistan	1	2,980,381	506,624	946,026	31,461
Tajikistan	2	2,132,997	334,989	624,271	20,883
Tajikistan	3	1,073,617	173,478	325,633	10,815
Tajikistan	4	2,558,537	423,792	791,528	26,444
Tunisia	1	4,156,065	1,421,020	2,652,235	88,352
Tunisia	2	2,993,243	934,482	1,744,116	58,014
Tunisia	3	1,711,642	556,561	1,039,231	34,718
Tunisia	4	3,578,598	1,197,556	2,241,228	74,471
Turkey	1	31,029,721	11,408,352	21,265,808	708,020
Turkey	2	23,141,898	7,967,972	14,847,504	495,400
Turkey	3	14,342,055	5,342,495	9,982,584	331,483
Turkey	4	27,262,980	9,879,608	18,427,322	614,391
Tanzania	1	19,439,045	2,891,250	5,398,276	180,023
Tanzania	2	14,683,607	2,076,716	3,872,581	129,155
Tanzania	3	7,943,080	1,196,375	2,231,612	74,240
Tanzania	4	17,106,352	2,514,752	4,689,457	156,668
Uganda	1	14,035,585	2,241,332	4,179,979	139,292
Uganda	2	10,795,699	1,767,713	3,302,957	109,887
Uganda	3	9,634,472	1,547,767	2,892,964	96,320
Uganda	4	12,625,197	2,044,288	3,816,800	126,941
Yemen, Rep.	1	8,978,940	1,189,341	2,223,059	74,108
Yemen, Rep.	2	6,443,446	752,402	1,407,659	46,809
Yemen, Rep.	3	3,134,621	389,461	723,167	23,985
Yemen, Rep.	4	7,619,709	969,123	1,816,361	60,463
South Africa	1	20,484,692	4,996,027	9,320,556	310,572
South Africa	2	15,232,037	3,438,007	6,410,413	213,792
South Africa	3	8,795,217	2,079,589	3,890,085	129,416
South Africa	4	17,929,453	4,284,329	7,988,411	266,130
Zambia	1	5,908,590	719,278	1,341,163	44,603
Zambia	2	4,464,221	514,500	966,758	32,043
Zambia	3	2,130,687	259,718	486,324	16,192
Zambia	4	5,177,648	620,035	1,154,007	38,561
Zimbabwe	1	4,572,304	991,499	1,852,342	61,684
Zimbabwe	2	3,484,742	817,788	1,524,035	50,875
Zimbabwe	3	3,275,359	779,773	1,450,086	48,299
Zimbabwe	4	4,110,615	926,563	1,728,563	57,740

Torres-Rueda S, *et al. BMJ Global Health* 2022; 6:e005759. doi: 10.1136/bmjgh-2021-005759

2. Health resource use and costing parameters and assumptions

2.1. Summary

We summarise the main parameters used in the estimates of health resources and costing. Further details and references are then provided in the following sections.

In summary, there are five steps in our calculations:

1. Calculation of unit costs per activity for three base countries: Ethiopia (low-income country or 'LIC'), Pakistan (lower-middle income country or 'lower-MIC') and South Africa (upper-middle income country or 'upper-MIC')
2. Extrapolation of unit costs in base countries to calculate unit costs across LICs, lower-MICs and upper-MICs
3. Calculation of total costs per country using country-specific unit costs, modelled data on the number of cases, hospitalisations and deaths, as well as other epidemiological and economic assumptions
4. Calculation of country-specific costs per capita, as well as costs per capita as a proportion of gross domestic product (GDP) per capita and various measures of health expenditure per capita

2.2 Calculation of unit costs per activity for three base countries

2.2.1 General Approach

A full economic costing was carried out over a one-year time horizon. Costs were constructed using a bottom-up ingredients-based technique. The costing was carried out from a health systems perspective and included both direct (e.g. medicines) and indirect costs (e.g. facility overheads). No above-service delivery costs were included.

The 76 countries chosen met three inclusion criteria: 1) classify as low-income, lower-middle income or upper-middle income by the World Bank (17), 2) be included in the list of 92 countries for which epidemiological modelling data was available from Pearson et al (2020) (2), and 3) have recent available GDP per capita (adjusted for PPP) data in order to carry out cost extrapolation between countries (17).

2.2.2 Intervention costs

We used official WHO guidance to identify areas related to critical preparedness, readiness and response actions for COVID-19 to define a set of interventions involved in a national response to the pandemic (18). We identified 6 priority areas of work and is further sub-divided into 13 activities.

- Emergency response mechanisms at the national level
- Risk communication and community engagement
- Case finding, contact tracing and management
- Surveillance
- Public health measures
- Case management

For the first five areas of work we considered only WHO guidance to define the resource use. For case management costs we assumed less resource-intensive activities thought to be more plausible in low- and middle-income settings (‘real-world’). Assumptions on ‘real world’ resource use were based on the clinical expertise of members of the research team and are detailed below.

Following this guidance on areas of work, we generated a list of activities for which we needed to estimate unit costs (see Table SM5). These unit costs were brought together with the COVID epidemiological model to estimate resource needs.

2.2.3 Table SM5: Activities and unit types

Activity	Unit Type
1.a. Emergency Response Mechanisms: National level	Per country per day
1.b. Emergency Response Mechanisms: Training of health staff	One-off per site
2. Risk communication & community engagement	Per country per day
3.a. Case finding, contact tracing and management: Contact tracing	Per person contacted
3.b. Case finding, contact tracing and management: Quarantine of contacts	Per person quarantined
4.a. Surveillance: Case notification	Per positive case
4.b. Surveillance: Reporting (national level)	Per country per week
5. Public health measures: Hygiene education	Per education campaign per month
6. Screening and diagnosis	Per person screened and tested
7.a. Case Management: Home-based care	Per person requiring home-based care
7.b. Case Management: Hospital-based (severe case)	Per day of hospitalisation (severe case)
7.c. Case Management: Hospital-based (critical case)	Per day of hospitalisation (critical case)
7.d. Case Management: Death	Per COVID-related death

2.2.4 Defining inputs, inputs quantities and input costs

In order to calculate a unit cost for each of the abovementioned activities, we used an ingredients-based costing to identify a series of input required. For each input we estimated quantities needed and a country-specific price per quantity (see Table SM6). The costs of each input were identified using a range of sources, according to availability of recent primary cost data and appropriateness of cost estimates to the COVID-19 pattern of care. More details can be found below.

To obtain yearly costs per country, the unit costs below were then multiplied by the number of country-specific units (see Table SM12 for more details).

Example:
In the case of Emergency Response Mechanisms: National level (1a) we aim to calculate a cost per day. We assumed that the three inputs required *per day* are: (i) 10 junior-level government officials, (ii) 10 senior-level government officials, as well as (iii) meeting space and equipment for those 20 people. The salary for one day of work for one junior-level government official in Ethiopia was estimated at US$12.27, for one senior-level government official at US$17.29 and the cost of one day's worth of space and equipment necessary for meetings was estimated at US$13.18 per person. We multiplied inputs by prices: (US$12.27 x 10) + (US$17.29 x 10) + (US$13.18 x 20), which equals US$559.26. This represents the cost per day of the emergency response mechanism at the national level.

In order to determine the annual costs per country, this number was then multiplied by the total number of working days, assumed to be 260 (see Table SM12).

2.2.5 Table SM6: Quantities and unit costs per input per activity per country

Component	Number of Units per Input	Unit Cost per Inputs		
		Ethiopia	Pakistan	South Africa
**1.a. Emergency Response Mechanisms: National level**				
Working day (junior level govt)	10	$12.27	$13.07	$194.66
Working day (senior level govt)	10	$17.29	$23.94	$256.72
Meeting/ training costs per person per day	20	$13.18	$20.44	$159.17
Total (per country per day):		*$559.26*	*$778.90*	*$7,697.16*
**1.b. Emergency Response Mechanisms: Training of health staff**				
Working day (health care workers)	250	$4.93	$10.43	$97.58
Working day (junior level govt)	10	$12.27	$13.07	$194.66
Working day (senior level govt)	1	$17.29	$23.94	$256.72
Meeting/ training costs per person per day	261	$13.18	$20.44	$159.17
Total (one-off per site):		*$4,813.58*	*$8,096.53*	*$68,141.36*
**2. Risk communication & community engagement**				
Working day (junior level govt)	3	$12.27	$13.07	$194.66
Working day (senior level govt)	2	$17.29	$23.94	$256.72
Media costs per day (office space)	1	$2.74	$4.58	$36.00
Total (per country per day):		*$74.14*	*$91.67*	*$1,133.44*
**3.a. Case finding, contact tracing and management: Contact tracing**				
Working day (junior level govt)	0.1	$12.27	$13.07	$194.66
Contact tracing household visit	0.33	$2.08	$3.02	$13.68
Contact tracing phone call	0.67	$2.34	$0.34	$3.31
Total (per person contacted):		*$3.48*	*$2.54*	*$26.23*
**3.b. Case finding, contact tracing and management: Quarantine of contacts**				
Working day (health care workers)	0.1	$4.93	$10.43	$97.58
Working day (junior level govt)	0.1	$12.27	$13.07	$194.66
Total (per person quarantined):		*$1.72*	*$2.35*	*$29.22*
**4.a. Surveillance: Case notification**				
Working day (health care workers)	0.1	$4.93	$10.43	$97.58
Working day (junior level govt)	0.1	$12.27	$13.07	$194.66
Total (per positive case):		*$1.72*	*$2.35*	*$29.22*
**4.b. Surveillance: Reporting (national level)**				
Working day (health care workers)	0.5	$4.93	$10.43	$97.58
Working day (junior level govt)	0.1	$12.27	$13.07	$194.66
Total (per country per week):		*$3.69*	*$6.52*	*$68.26*
**5. Public health measures: Hygiene education**				
Working day (junior level govt)	2	$12.27	$10.43	$97.58
Working day (senior level govt)	1	$17.29	$13.07	$194.66
Media costs per day	1	$2.74	$4.58	$36.00
Total (per education campaign per month):		*$44.58*	*$38.51*	*$425.83*
**6. Screening and diagnosis**				
Ambulance trip	0.0001	$4.80	$9.51	$60.41
Isolation pod/ diagnostic visit	2	$0.49	$0.49	$7.97
Outpatient visit oral history	1	$3.57	$0.47	$8.02
Outpatient visit physical exam	1	$3.57	$0.47	$8.02
Outpatient visit specimen collection	1	$4.88	$1.09	$17.15
COVID19 test (PCR)	1	$23.98	$23.98	$23.98
Total (per person screened and tested):		*$36.97*	*$26.98*	*$73.12*

Component	Number of Units per Input	Unit Cost per Inputs		
		Ethiopia	Pakistan	South Africa
**7.a. Case Management: Home-based care**				
Home-based care bed-day	5	$0.94	$0.61	$11.65
Community-based care via clinicians visit	2	$9.11	$4.71	$44.16
Total (per person requiring home-based care):		*$22.90*	*$12.45*	*$146.57*
**7.b. Case Management: Hospital-based (severe case)**				
Inpatient ward bed-day (severe)	1	$29.90	$31.54	$96.66
Diagnostics				
Pulse oximetry	0.125	$0.00	$0.00	$0.00
Chest X-ray	0.125	$27.35	$2.79	$21.86
Full blood count	0.125	$2.37	$2.29	$24.28
Blood urea and electrolyte test	0.125	$4.20	$2.53	$2.87
C-reactive protein test	0.125	$2.34	$0.32	$5.15
HIV test	0.125	$4.38	$3.87	$17.13
COVID19 test (PCR)	0	$23.98	$23.98	$ 23.98
Malaria test	0.125	$0.19	$0.19	$0.19
Haemoglobin test	0.125	$2.29	$2.29	$2.29
Total (per day of hospitalisation (severe case)):		*$35.29*	*$33.32*	*$105.88*
**7.c. Case Management: Hospital-based (critical case)**				
Inpatient ward bed-day (critical)	0.33	30.65	32.29	97.41
ITU bed-day	0.67	$104.48	$101.99	$662.71
Additional resourcing per COVID-related complication				
Acute respiratory distress syndrome (ARDS)	0.47	$22.46	$22.46	$22.46
Acute kidney injury days	0.04	$10.60	$10.60	$10.60
Acute cardiac injury days	0.06	$46.25	$46.25	$46.25
Liver dysfunction days	0.06	$89.32	$89.32	$89.32
Pneumothorax days	0.01	$6.66	$6.77	$7.02
Hospital-acquired pneumonia days	0.05	$18.85	$18.85	$18.85
Bacteraemia days	0.01	$32.55	$32.55	$32.55
Urinary tract infection days	0.01	$9.03	$9.03	$9.03
Septic shock days	0.05	$0.64	$0.67	$0.75
Diagnostics				
Pulse oximetry	10	$0.00	$0.00	$0.00
Chest X-ray	10	$27.35	$2.79	$21.86
Full blood count	10	$2.37	$2.29	$24.28
Blood urea and electrolyte test	10	$4.20	$2.53	$2.87
C-reactive protein test	10	$2.34	$0.32	$5.15
Venous blood gas test	10	$4.23	$4.23	$4.23
HIV test	0.1	$4.38	$3.87	$17.13
COVID19 test (PCR)	0	$23.98	$23.98	$23.98
Malaria test	0.1	$0.19	$0.19	$0.19
Haemoglobin test	0.1	$2.29	$2.29	$2.29
Total (per day of hospitalisation (critical)):		$505.56	$221.18	$1,081.94
**7.d. Case Management: Death**				
Body Bag	1	$64.52	$64.52	$64.52
Total (per COVID-related death):		$64.52	$64.52	$64.52

2.2.6 Input quantities

Activities 1-6:
Quantities of working days required for planning and management and communication were estimated from expert consultation as part of the Disease Control Priorities 3-Universal Health Coverage (DCP3-UHC) project (19). For case finding, surveillance and diagnostic activities, quantities were estimated based on requirements for similar activities for tuberculosis (TB) such as contact tracing from the VALUE TB study and previous studies in South Africa (more below) (20, 21).

Activities 7:
The number of days per patient in general ward and in ICU was set at 8 and 10 respectively and was set to match the assumptions in the epidemiological model (2, 14, 22). Following expert clinician advise we assumed that one-third of critical patient bed days would be treated the general ward and two-thirds in the ICU.

The likelihood of additional COVID-related complications (per day) were estimated using evidence on the clinical course of COVID from patients in Wuhan, China (23), as were assumptions on the duration of symptoms (24, 25). The number of diagnostic tests per hospitalisation was carried out in consultation with expert clinicians in essential critical care.

2.2.7 Input unit costs

2.2.7.1 Estimation of non-bed-day costs (Pakistan)

An ingredients-based approach was used to calculate most of the service costs and prices for Pakistan. The data used was collected as part of the Disease Control Priorities 3-Universal Health Coverage (DCP3-UHC) project (19). For other countries primary data from the TB studies was used (see below).

For Pakistan, staff-related costs were constructed using federal-level pay scales. For most outputs, the number of minutes of staff required per activity were estimated via expert opinion obtained from clinicians working in the Health Planning, System Strengthening & Information Analysis Unit (HPSIU) in the Ministry of National Health Services Regulations and Coordination of Pakistan. For outputs where this was unavailable, health economists agreed a plausible assumed value.

Drug regimens were costed using resource use data obtained through expert opinion (HPSIU) and a number of price sources. An assessment of strengths and weaknesses of different price sources was conducted and hierarchy of sources was established. The primary source of price data was the Sindh Health Department Procurement Price list. If a price was unavailable, the Federal Wholesale Price List for Generic Medicines was used as a second option. As a last resort, private sector market prices were used.

Cost on supplies and equipment were similarly constructed. Resource use was determined through expert opinion (HPSIU) and price source hierarchy established. The primary source

was the Medical Emergency Resilience Fund 2019-2020, and a secondary source was private sector market prices.

For all countries, for additional diagnostic and radiology costs (beyond those available from the TB data) were estimated using available literature and market prices. We assessed strengths and weaknesses of different price sources. For example, we used the 'Costing and Pricing of Services in Private Hospitals of Lahore: Summary Report' as our primary source as it contained a methodological appendix that suggested that an ingredients-based approach consistent with ours was followed. If some prices were unavailable we used user fees from the Pakistan Institute of Medical Sciences, procurement prices from the Medical Emergency Resilience Fund procurement prices and user fees from the Aga Khan University Hospital.

Space costs were estimated using data from budget documents from the Federal government (Islamabad Capital Territory Health Infrastructure PC-1).

Oxygen therapy costs per bed-day were calculated by estimating the number of cylinders consumed in 24 hours at different flow rates, assumed to be 10L per minute in the general ward and 30L per minute in the ICU. Cylinder duration (hours) was estimated by dividing pressure by the number of litres per minute, assuming a standard cylinder size of 4.6kg, filled at 1,900 psi pressure (26). Cost per cylinder was obtained from the South African online catalogue of a manufacturer that is active in both South Africa and Pakistan (27).

2.2.7.2 Estimation of non-bed-day costs (Ethiopia and South Africa)

For Ethiopia and South Africa the main source of cost data was the VALUE TB study (20, 21). Cost data were collected from a health provider perspective to estimate the economic costs of TB-related health services. Full costs of health services were estimated. Cost data collection was retrospective, over a one-year period to minimize the risk of bias due to seasonality. Resource use was measured in the VALUE TB study using both top-down and bottom-up methods wherever possible, to allow for comparison. The costs included in the current cost model reflected an average of top-down and bottom-up costs by site. For South Africa, we also used primary data from the XTEND trial (nurses and lay health workers) (28).

Some of the COVID-19 interventions were outside the scope of the VALUE TB and XTEND studies. Values for which a primary unit cost was partially or entirely unavailable from Value TB are listed below. For these interventions, resource use data from Pakistan was used with local Ethiopian or South African prices.

- **Planning & coordination activities:** Working day (mid-level facility); Working day (junior level govt); Working day (senior level govt); Meeting/ training costs per day; Media costs per day; Health hotline (day)
- **Infection control:** Ambulance trip; Isolation pod/ diagnostic visit; Deep clean
- **Home-based care:** Home-based care bed-day; Community-based care via GP
- **Inpatient treatment:** Inpatient ward bed-day including PPE (normative scenario); ICU bed-day, including PPE (normative scenario); Severe case ward bed-day,

including PPE; Critical case ward bed-day, including PPE; ITU bed-day ('real-world scenario'); Body disposal
- **Additional resourcing per COVID related complication:** Acute respiratory distress syndrome (ARDS); Acute kidney injury; Acute cardiac injury; Liver dysfunction; Pneumothorax; Hospital-acquired pneumonia; Bacteraemia; Urinary tract infection; Septic shock
- **Investigations (lab tests):** Pulse oximetry; Venous blood gas; Mid-stream urine test; COVID-19 confirmatory lab test (PCR); Malaria; Haemoglobin

2.2.7.3 Price adjustments

Where Pakistan health care inputs were applied to other settings, we classified them as tradeable or non-tradeable. For tradable inputs, where country-specific price estimates were not available from primary data or from the published literature, the estimate from Pakistan was applied to other countries. For non-tradable inputs, the estimate from Pakistan was adjusted by an amount reflecting the difference in the two countries' GDP (adjusted for purchasing power parity, or PPP) (see Table SM7). The rationale behind this approach is that, while exchange rate may be influenced by government policy, PPP seeks to equalise the purchasing power of different currencies and, as such, may better reflect differentials in non-tradable prices across countries. More details on this method of price adjustment can be found in Section 2.3. Staff costs did not need to be extrapolated as we had country-specific salary information for the three countries.

2.2.7.4 Table SM7: Relative GDP adjustment factors

Country	Exchange rate (US$)	GDP per capita by country (US$ PPP)	Relative GDP (PPP): Pakistan	Relative GDP (PPP): South Africa	Relative GDP (PPP): Ethiopia
Pakistan	155.00	5,567.06	1.00	0.41	2.75
South Africa	32.26	2,022.14	2.46	1.00	6.77
Kenya	76.92	7,762.88	0.62	0.25	1.71
India	104.17	3,467.56	1.39	0.57	3.84
Ethiopia	16.95	13,686.88	0.36	0.15	1.00

2.2.7.5 Estimation of bed-day costs (all countries)

We took an ingredients-based approach to estimating the costs of general ward and ICU ward bed days, as these were major cost drivers in our cost model. We estimated the plausible number of nursing hours per bed day in an LMIC setting through consultation with members of the research team who have expertise in critical care in LMICs. In ICU the assumption of nurse-to-patient ratio would be 1:1; in the general ward the ratio would be 1:6 during the day time and 1:20 in the night.

To understand the full range of inputs required we obtained the underlying costing data set provided by the authors of a recent costing of hospital-based care (29). The paper reports the results of a detailed activity-based costing in a hospital in Karachi, disaggregated by phase of care. We used the cost data for the ward stay phase, removing any supplies or equipment specific to the surgery, to estimate the average generic costs of a bed-day.

All bed-day costs were compared to and validated against available country-specific estimates from the published literature and from ongoing research and WHO CHOICE (see Table SM8). Rapid literature searches were conducted on the Medline, Embase and EconLit databases on 8- 9 April 2020 to identify records reporting on the costs of ICU care in each of the study countries.

We estimated the additional costs of ICU beds compared to standard hospital beds using an ingredients-based approach to cost the equipment and supplies not present in standard hospital wards. We used the procurement price of equipment and assumed depreciation over ten (ventilators and suction pumps) or five years (all other equipment). Supply costs included central and arterial lines, ventilator tubing, and sedatives.

2.2.7.6 COVID-19 specific costs

Finally, we calculated costs of supplies and inputs specific to COVID-19. Personal protective equipment (PPE) per health worker per day (see Table SM8) was calculated and allocated a cost per PPE per minute to clinical staff. We also calculated costs of hygiene per bed day (see Table SM9). We estimated the costs of PPE and hygiene supplies using a list of necessary supplies from a COVID-related budget from the Ministry of Health of Pakistan, which included local prices sourced by the Aga Kahn University. This was complemented for other countries using the WHO's Essential Supplies Forecasting Tool (ESFT) (30). We divided supplies into single-use and disposable. We determined plausible quantities and useful life for supplies following clinical guidelines and expert opinion.

Oxygen supplementation therapy is the main form of treatment for COVID-19. There are different methods of oxygen delivery which utilise different types of supplies, equipment and require different average levels of oxygen flow. We calculated costs for 6 types of oxygen delivery techniques and assumed a distribution across severe and critical patients according to members of our research team with clinical expertise in critical care in LMICs. Table SM10 shows the assumptions used in our model and how they differ from normative standards.

2.2.7.7 Table SM8: PPE costs per general ward bed day and per ICU bed day

**Supply**	**Price US$**	**Useful life (days)**	**Quantity per day**	**Total per member of staff per day US$**	**Assumptions**
**PPE for General Ward**					
Single Use					
Surgical Gowns	0.20	1	1	0.20	
Nitrile Gloves	0.05	1	10	0.45	
Latex Gloves	0.04	1	10	0.39	
Disposable Head	0.03	1	4	0.10	
Shoe Covers	0.02	1	4	0.06	
Surgical Masks	0.08	1	10	0.77	
Reusable					
Goggles	11.61	90	1.5	0.19	Assuming half a day for washing
Gum Boots	19.35	90	1.5	0.32	Assuming half a day for washing
**TOTAL**				**2.50**	
**PPE for ICU**					
Single Use					
N-95 Masks	0.84	1	4	3.35	
Disposable apron	0.20	1	1	0.20	
Nitrile Gloves	0.05	1	10	0.45	
Latex Gloves	0.04	1	10	0.39	
Disposable Head	0.03	1	4	0.10	
Shoe Covers	0.02	1	4	0.06	
Surgical Masks	0.08	1	10	0.77	
Reusable					
Face Shields	27.81	5	1.5	8.34	Assuming half a day for washing
Goggles	11.61	90	1.5	0.19	Assuming half a day for washing
Gum Boots	19.35	90	1.5	0.32	Assuming half a day for washing
**TOTAL**				**14.19**	

2.2.7.8 Table SM9: Hygiene costs per general ward and ICU bed day

**Supply**	**Price US$**	**Useful life (days)**	**Quantity per day**	**Total per ICU bed per day US$**	**Assumptions**
Single Use					
Hand Sanitizers	47.97	1	0.05	2.40	100ml use per day, price assumed to refer to bottle of 2000ml
Biohazard Bags	0.23	1	1	0.23	
Disposable bed sheets	1.94	1	1	1.94	
Disposable Tissue Boxes	0.65	1	1	0.65	1 box per day, price assumed to refer to 1 box
Disposable Tissue rolls	0.35	1	1	0.35	1 roll per day, price assumed to refer to 1 roll
Disinfectants (1L Dettol)	3.23	1	0.25	0.81	250ml used per day, price refers to bottle of 1000ml
Liquid Soaps (250ml Dettol bottles)	1.74	1	0.2	0.35	50ml used per day, price refers to bottle of 250ml
Ethanol (1L bottles)	16.13	1	0.1	1.61	100ml used per day, price refers to bottle of 1000ml
Liquid Bleach	2.58	1	0.25	0.65	250ml used per day, price assumed to refer to bottle of 1000ml
Reusable					
Waste Bins	15.03	90	1	0.17	
Mackintosh bed sheets	9.68	90	1	0.11	
Mops	2.58	90	1	0.03	
Dusters	0.32	90	1	0.00	
TOTAL				9.28	

2.2.7.9 Table SM10: Oxygen supplementation assumptions

	**Normative recommendations**		**'Real-world' scenario**		
	**Severe case**	**Critical case**	**Severe case**	**Critical case**	
	Severe pneumonia (15% of COVID cases)	Acute respiratory distress syndrome (5% of COVID cases)	Severe pneumonia (15% of COVID cases)	Acute respiratory distress syndrome (5% of COVID cases)	
	**General ward**	**ICU**	**General ward**	**General ward only**	**ICU**
Supplemental oxygen management type					
% ventilator	0%	100%	0%	0%	50%
% CPAP	0%	0%	0%	0%	25%
% high-flow nasal cannula	0%	0%	0%	0%	25%
% non-rebreather mask	25%	0%	25%	100%	0%
% nasal cannula	50%	0%	50%	0%	0%
% high-concentration mask	25%	0%	25%	0%	0%
% Patients in pathway	**100%**	**100%**	**100%**	**33%**	**67%**

2.3 Extrapolation of unit costs in base countries to calculate unit costs across LICs, Lower-MICs and Upper-MICs

We used the unit costs obtained in our three base countries to extrapolate unit costs to other LICs, Lower-MICs and Upper-MICs. We grouped countries according to income group. Costs for LICs were extrapolated using unit costs from Ethiopia, costs for LMICs were extrapolated from the unit costs from Pakistan, and those for UMICs from the unit costs from South Africa.

In order to carry out the extrapolation, each cost ingredient for each of the unit costs was classified as a tradeable good, non-tradeable good, or staff cost.

Tradeable goods are generally defined as those that can easily be traded in the international market and include goods such as medical or other supplies and medications. The unit costs for our three base countries were initially converted from each local currency into 2019 US$ using market exchange rates. To convert the tradeable good from the base country (e.g. Ethiopia) to a 'second' country (e.g. Afghanistan) we apportioned the percentage of the unit cost that was composed of tradeable goods in 2019 US$ from the base country to the second country.

Non-tradeable goods include buildings, heavy machinery, and other equipment. To convert these costs from a base country to a second country we used purchasing power parity (PPP) conversion rates. We multiplied the proportion of the unit cost that was defined as non-tradeable (in 2019 US$) by the ratio of the GDP per capita (adjusted for PPP) of the second county, divided by the GDP per capita (adjusted for PPP) of the base country. Data on GDP per capita (adjusted for PPP) can be found in the World Bank database (17).

To convert staff costs from a base country to a second country we used conversion rates from Serje et al (2018) (31). Serje et al (2018) use regression analysis on a dataset containing wages from health workers of different skill levels for 193 countries in order to predict wages by country income level relative to GDP per capita. We used the multiples per GDP per capita presented in the paper in order to convert the staff wages from the base country to the second country. See Table SM11.

2.3.1 Table SM11: Health worker earnings as a multiple of GDP per capita

**World bank income categories**	**Health worker cadre**	**Average earnings index (multiple of GDP per capita)**
High-income countries	Physicians	1.9
	Nurses and midwives	1.5
	Other health workers	0.9
Upper-middle income countries	Physicians	2.7
	Nurses and midwives	2.2
	Other health workers	1.3
Lower-middle income countries	Physicians	5.1
	Nurses and midwives	4.2
	Other health workers	2.4
Lower-income countries	Physicians	7.8
	Nurses and midwives	6.4
	Other health workers	3.7
Global	Physicians	4.4
	Nurses and midwives	3.6
	Other health workers	2.1

2.4 Calculation of country-specific number of units per activity

The unit cost in each of the 76 countries was used to calculate the total costs per activity per country. Table SM12 shows the quantities that those unit costs were multiplied by in order to calculate the total costs per country, as well as their justification and source.

2.4.1 Table SM12: Number of country-specific units per activity

Activity	Unit Type	Quantities per country	Value	Source
1.a. Emergency Response Mechanisms: National level	Per country per day	Number of working days per year	260	Assumption
1.b. Emergency Response Mechanisms: Training of health staff	One-off per site	Total number of clinical sites	Variable per Country	Calculated by assuming one site for every 200 hospital beds available in the country (32)
2. Risk communication & community engagement	Per country per day	Number of calendar days per year	365	N/A
3.a. Case finding, contact tracing and management: Contact tracing	Per person contacted	Total number of COVID19 cases *	Variable by country	See Table SM4
		% cases that are symptomatic *	69%	(33)
		% of symptomatic cases tested *	10%	Assumption
		Average number of contacts per COVID19-positive case	7	(34)
3.b. Case finding, contact tracing and management: Quarantine of contacts	Per person quarantined	Total number of COVID19 cases *	Variable by country	See Table SM4
		% cases that are symptomatic *	69%	(33)
		% of symptomatic cases tested *	10%	Assumption
		Average number of contacts per COVID19-positive case	7	(34)
4.a. Surveillance: Case notification	Per positive case	Total number of COVID19 cases *	Variable per Country	See Table SM4
		% cases that are symptomatic *	69%	(33)

Activity	Unit Type	Quantities per country	Value	Source
		% of symptomatic cases tested *	10%	Assumption
4.b. Surveillance: Reporting (national level)	Per country per day	Total number of clinical sites *	Variable per Country	Calculated by assuming one site for every 200 hospital beds available in the country (32)
		Weeks per year	52	N/A
5. Public health measures: Hygiene education	Per education campaign	Months per year	12	N/A
6. Screening and diagnosis‡	Per person screened and tested	(Total number of COVID19 cases *	Variable per Country	See Table SM4
		% of cases requiring hospitalisation *	18.50%	(2, 14)
		Number of people tested per positive case) +	11.31	See Table SM13
		(Total number of COVID19 cases *	Variable per Country	See Table SM4
		% cases that are symptomatic *	69%	(33)
		% of symptomatic cases tested *	10%	Assumption
		Number of people tested per positive case)	11.31	See Table SM13
7.a. Case Management: Home-based care‡	Per person requiring home-based care	Proportion of borderline mild-to-severe cases	10%	Assumption
7.b. Case Management: Hospital-based (severe case) ‡	Per day of hospitalisatio n (severe case)	Average number of days of hospitalisation for severe cases	8	(2, 22)
7.c. Case Management: Hospital-based (critical case) ‡	Per day of hospitalisatio n (critical case)	Average number of days of hospitalisation for critical cases	10	(2, 14)
7.d. Case Management: Death‡	Per COVID-related death	Total number of deaths from COVID19	Variable per Country	See Table SM4

Note: Scenario 1 modelled an unmitigated epidemic. Therefore, only activities marked with ‡ were included in calculating the costs for Scenario 1. Scenarios 2-4 included costs in all the activities mentioned in Table SM13.

2.4.2. Table SM13: Test positivity rate by country and average

Country	% of positive tests	Source
South Africa	0.169	(35)
Kenya	0.103	(34)
Ethiopia	0.0739	(36)
India	0.0612	(37)
Pakistan	0.0351	(38)
**Average**	**0.08844**	

2.5 Country-specific per capita costs and per capita costs as a proportion of gross domestic product (GDP) per capita and other measures of health expenditure per capita

Total costs per country were used to calculate the COVID-19-related costs per capita per country per scenario by dividing the total costs by the population of the country (17). The cost per capita was then calculated as a proportion of GDP per capita (nominal) (17) and three measures of health expenditure per capita (39): 1) total health expenditure including out-of-pocket payments, 2) total health expenditure excluding out-of-pocket payments, and 3) government health spending per capita. Data on GDP per capita and health expenditure per capita per country can be found in Table SM14.

Torres-Rueda S, *et al. BMJ Global Health* 2022; 6:e005759. doi: 10.1136/bmjgh-2021-005759

2.5.1 Table SM14: Population, GDP and health spending per country

Country	Country income classification	Total population per country	Gross Domestic Product per Capita (Nominal) (US$)	Gross Domestic Product per Capita (PPP) (US$)	Total Health Spending per Capita (including out-of-pocket spending) (US$)	Total Health Spending per Capita (excluding out-of-pocket spending) (US$)	Government Health Spending per Capita (US$)
Afghanistan	LIC	37,172,386	$521	$1,955	$57	$102	$3
Albania	Upper-MIC	2,866,376	$5,269	$13,364	$272	$429	$112
Algeria	Upper-MIC	42,228,429	$4,115	$15,482	$260	$341	$176
American Samoa	Upper-MIC	55,465	$11,467	N/A	N/A	N/A	N/A
Angola	Lower-MIC	30,809,762	$3,432	$6,452	$95	$129	$42
Argentina	Upper-MIC	44,494,502	$11,684	$20,611	$955	$1,106	$711
Armenia	Upper-MIC	2,951,776	$4,212	$10,343	$359	$648	$59
Azerbaijan	Upper-MIC	9,942,334	$4,721	$18,044	$268	$480	$54
Bangladesh	Lower-MIC	161,356,039	$1,698	$4,372	$34	$59	$6
Belarus	Upper-MIC	9,485,386	$6,290	$19,995	$318	$432	$195
Belize	Upper-MIC	383,071	$4,885	$8,648	$304	$373	$201
Benin	LIC	11,485,048	$902	$2,425	$30	$44	$6
Bhutan	Lower-MIC	754,394	$3,243	$10,168	$91	$110	$68
Bolivia	Lower-MIC	11,353,142	$3,549	$7,873	$213	$273	$140
Bosnia and Herzegovina	Upper-MIC	3,323,929	$6,066	$14,624	$444	$571	$314
Botswana	Upper-MIC	2,254,126	$8,259	$18,616	$380	$400	$212
Brazil	Upper-MIC	209,469,333	$8,921	$16,096	$1,016	$1,458	$338
Bulgaria	Upper-MIC	7,024,216	$9,273	$21,960	$612	$906	$310
Burkina Faso	LIC	19,751,535	$715	$1,985	$41	$54	$16
Burundi	LIC	11,175,378	$272	$744	$18	$24	$5
Cabo Verde	Lower-MIC	543,767	$3,635	$7,454	$159	$200	$90
Cambodia	Lower-MIC	16,249,798	$1,510	$4,361	$78	$123	$17
Cameroon	Lower-MIC	25,216,237	$1,534	$3,785	$64	$109	$9
Central African Republic	LIC	4,666,377	$476	$860	$16	$23	$2
Chad	LIC	15,477,751	$728	$1,968	$32	$51	$6
China	Upper-MIC	1,392,730,000	$9,771	$18,237	$398	$541	$231
Colombia	Upper-MIC	49,648,685	$6,668	$15,013	$340	$409	$216
Comoros	Lower-MIC	832,322	$1,415	$2,913	$59	$102	$9
Congo, Dem. Rep.	LIC	84,068,091	$562	$932	$21	$28	$3
Congo, Rep.	Lower-MIC	5,244,363	$2,148	$5,662	$70	$105	$30
Costa Rica	Upper-MIC	4,999,441	$12,027	$17,671	$889	$1,086	$664
Cote d'Ivoire	Lower-MIC	25,069,229	$1,716	$4,207	$68	$95	$17
Cuba	Upper-MIC	11,338,138	$8,822	N/A	$971	$1,071	$870
Djibouti	Lower-MIC	958,920	$3,083	N/A	$70	$88	$32
Dominica	Upper-MIC	71,625	$7,691	$11,130	$419	$542	$269
Dominican Republic	Upper-MIC	10,627,165	$8,051	$17,748	$414	$599	$189
Ecuador	Upper-MIC	17,084,357	$6,345	$11,734	$505	$709	$258
Egypt, Arab Rep.	Lower-MIC	98,423,595	$2,549	$12,412	$131	$212	$38
El Salvador	Lower-MIC	6,420,744	$4,058	$8,332	$294	$374	$189
Equatorial Guinea	Upper-MIC	1,308,974	$10,262	$22,744	$281	$486	$66
Eritrea	LIC	N/A	$811	N/A	$30	$48	$9
Eswatini	Lower-MIC	1,136,191	$4,146	$10,638	$221	$242	$153
Ethiopia	LIC	109,224,559	$772	$2,022	$28	$38	$8
Fiji	Upper-MIC	883,483	$6,267	$10,879	$180	$217	$115
Gabon	Upper-MIC	2,119,275	$7,953	$17,876	$220	$270	$142

Country	Country income classification	Total population per country	Gross Domestic Product per Capita (Nominal) (US$)	Gross Domestic Product per Capita (PPP) (US$)	Total Health Spending per Capita (including out-of-pocket spending) (US$)	Total Health Spending per Capita (excluding out-of-pocket spending) (US$)	Government Health Spending per Capita (US$)
Gambia, The	LIC	2,280,102	$716	$2,612	$21	$26	$4
Georgia	Upper-MIC	3,731,000	$4,717	$12,005	$308	$479	$113
Ghana	Lower-MIC	29,767,108	$2,202	$4,747	$68	$93	$26
Grenada	Upper-MIC	111,454	$10,640	$15,558	$516	$815	$213
Guatemala	Upper-MIC	17,247,807	$4,549	$8,462	$241	$370	$90
Guinea	LIC	12,414,318	$879	$2,505	$37	$56	$5
Guinea-Bissau	LIC	1,874,309	$778	$1,799	$39	$53	$17
Guyana	Upper-MIC	779,004	$4,979	$8,641	$192	$260	$113
Haiti	LIC	11,123,176	$868	$1,867	$38	$53	$6
Honduras	Lower-MIC	9,587,522	$2,500	$5,139	$200	$289	$92
India	Lower-MIC	1,352,617,328	$2,010	$7,763	$63	$103	$16
Indonesia	Lower-MIC	267,663,435	$3,894	$13,080	$112	$153	$50
Iran, Islamic Rep.	Upper-MIC	81,800,269	$5,628	N/A	$415	$577	$226
Iraq	Upper-MIC	38,433,600	$5,834	$17,436	$153	$272	$32
Jamaica	Upper-MIC	2,934,855	$5,354	$9,327	$296	$363	$179
Jordan	Upper-MIC	9,956,011	$4,242	$9,479	$224	$286	$141
Kazakhstan	Upper-MIC	18,276,499	$9,813	$27,880	$262	$355	$154
Kenya	Lower-MIC	51,393,010	$1,711	$3,468	$66	$85	$24
Kiribati	Lower-MIC	115,847	$1,625	$2,294	$188	$188	$116
Korea, Dem. People's Rep.	LIC	25,549,819	N/A	N/A	N/A	N/A	N/A
Kosovo	Upper-MIC	1,845,300	$4,302	$11,348	N/A	N/A	N/A
Kyrgyz Republic	Lower-MIC	6,315,800	$1,281	$3,885	$73	$115	$28
Lao PDR	Lower-MIC	7,061,507	$2,542	$7,440	$55	$81	$18
Lebanon	Upper-MIC	6,848,925	$8,270	$13,081	$662	$875	$345
Lesotho	Lower-MIC	2,108,132	$1,299	$3,219	$86	$102	$55
Liberia	LIC	4,818,977	$677	$1,309	$68	$101	$10
Libya	Upper-MIC	6,678,567	$7,242	$20,764	N/A	$115	N/A
Madagascar	LIC	26,262,368	$528	$1,891	$24	$30	$11
Malawi	LIC	18,143,315	$389	$1,311	$30	$33	$8
Malaysia	Upper-MIC	31,528,585	$11,373	$31,782	$362	$497	$182
Maldives	Upper-MIC	515,696	$10,331	$15,308	$1,048	$1,248	$760
Mali	LIC	19,077,690	$900	$2,317	$30	$40	$9
Marshall Islands	Upper-MIC	58,413	$3,788	$3,989	$851	$928	$448
Mauritania	Lower-MIC	4,403,319	$1,189	$4,151	$47	$71	$17
Mauritius	Upper-MIC	1,265,303	$11,239	$23,751	$553	$819	$244
Mexico	Upper-MIC	126,190,788	$9,673	$19,845	$462	$648	$241
Micronesia, Fed. Sts.	Lower-MIC	112,640	$3,568	$3,553	$387	$397	$108
Moldova	Lower-MIC	3,545,883	$3,227	$7,272	$171	$250	$84
Mongolia	Lower-MIC	3,170,208	$4,122	$13,800	$141	$191	$80
Montenegro	Upper-MIC	622,345	$8,844	$20,690	$532	$660	$399
Morocco	Lower-MIC	36,029,138	$3,238	$8,587	$171	$255	$80
Mozambique	LIC	29,495,962	$499	$1,460	$19	$21	$10
Myanmar	Lower-MIC	53,708,395	$1,326	$6,674	$62	$108	$12
Namibia	Upper-MIC	2,448,255	$5,931	$11,102	$403	$434	$249
Nauru	Upper-MIC	12,704	$9,889	$16,504	$1,012	$1,024	$615
Nepal	LIC	28,087,871	$1,034	$3,090	$45	$71	$8

Country	Country income classification	Total population per country	Gross Domestic Product per Capita (Nominal) (US$)	Gross Domestic Product per Capita (PPP) (US$)	Total Health Spending per Capita (including out-of-pocket spending) (US$)	Total Health Spending per Capita (excluding out-of-pocket spending) (US$)	Government Health Spending per Capita (US$)
Nicaragua	Lower-MIC	6,465,513	$2,029	$5,534	$188	$249	$115
Niger	LIC	22,442,948	$414	$1,063	$23	$36	$6
Nigeria	Lower-MIC	195,874,740	$2,028	$5,991	$79	$139	$10
North Macedonia	Upper-MIC	2,082,958	$6,084	$16,359	$328	$444	$208
Pakistan	Lower-MIC	212,215,030	$1,482	$5,567	$40	$65	$11
Papua New Guinea	Lower-MIC	8,606,316	$2,730	$4,336	$55	$59	$39
Paraguay	Upper-MIC	6,956,071	$5,822	$13,600	$327	$451	$169
Peru	Upper-MIC	31,989,256	$6,941	$14,418	$316	$406	$203
Philippines	Lower-MIC	106,651,922	$3,103	$8,951	$129	$199	$41
Romania	Upper-MIC	19,473,936	$12,301	$28,206	$476	$575	$372
Russian Federation	Upper-MIC	144,478,050	$11,289	$27,147	$469	$659	$267
Rwanda	LIC	12,301,939	$773	$2,252	$48	$51	$16
Samoa	Upper-MIC	196,130	$4,183	$6,484	$227	$254	$173
Sao Tome and Principe	Lower-MIC	211,028	$2,001	$3,419	$105	$120	$42
Senegal	Lower-MIC	15,854,360	$1,522	$3,783	$53	$80	$18
Serbia	Upper-MIC	6,982,084	$7,247	$17,435	$494	$695	$287
Sierra Leone	LIC	7,650,154	$534	$1,602	$86	$122	$10
Solomon Islands	Lower-MIC	652,858	$2,138	$2,423	$106	$111	$74
Somalia	LIC	15,008,154	$315	N/A	N/A	N/A	N/A
South Africa	Upper-MIC	57,779,622	$6,374	$13,687	$428	$461	$230
South Sudan	LIC	10,975,920	$1,120	N/A	N/A	N/A	N/A
Sri Lanka	Upper-MIC	21,670,000	$4,102	$13,474	$153	$230	$66
St. Lucia	Upper-MIC	181,889	$10,566	$13,881	$490	$728	$206
St. Vincent and the Grenadines	Upper-MIC	110,210	$7,361	$12,288	$250	$302	$192
Sudan	Lower-MIC	41,801,533	$977	$4,759	$152	$264	$30
Suriname	Upper-MIC	575,991	$6,234	$15,510	$356	$434	$247
Syrian Arab Republic	LIC	16,906,283	$2,033	N/A	N/A	$36	N/A
Tajikistan	LIC	9,100,837	$827	$3,450	$56	$92	$16
Tanzania	LIC	56,318,348	$1,051	$3,227	$35	$43	$14
Thailand	Upper-MIC	69,428,524	$7,274	$19,051	$222	$249	$173
Timor-Leste	Lower-MIC	1,267,972	$2,036	$7,658	$80	$87	$45
Togo	LIC	7,889,094	$679	$1,774	$39	$58	$8
Tonga	Upper-MIC	103,197	$4,364	$6,420	$203	$225	$134
Tunisia	Lower-MIC	11,565,204	$3,448	$12,503	$257	$359	$145
Turkey	Upper-MIC	82,319,724	$9,370	$28,069	$469	$546	$368
Turkmenistan	Upper-MIC	5,850,908	$6,967	$19,304	$423	$745	$78
Tuvalu	Upper-MIC	11,508	$3,701	$4,050	$507	$511	$429
Uganda	LIC	42,723,139	$643	$2,038	$38	$53	$6
Ukraine	Lower-MIC	44,622,516	$3,095	$9,233	$141	$218	$60
Uzbekistan	Lower-MIC	32,955,400	$1,532	$8,556	$135	$206	$62
Vanuatu	Lower-MIC	292,680	$3,124	$3,221	$110	$119	$59
Venezuela, RB	Upper-MIC	28,870,195	$16,054	N/A	N/A	$446	N/A
Vietnam	Lower-MIC	95,540,395	$2,567	$7,448	$123	$178	$58

Country	Country income classification	Total population per country	Gross Domestic Product per Capita (Nominal) (US$)	Gross Domestic Product per Capita (PPP) (US$)	Total Health Spending per Capita (including out-of-pocket spending) (US$)	Total Health Spending per Capita (excluding out-of-pocket spending) (US$)	Government Health Spending per Capita (US$)
West Bank and Gaza	Lower-MIC	4,569,087	$3,199	$5,158	N/A	N/A	N/A
Yemen, Rep.	LIC	28,498,687	$944	$2,575	$72	$130	$7
Zambia	Lower-MIC	17,351,822	$1,540	$4,224	$57	$63	$22
Zimbabwe	Lower-MIC	14,439,018	$2,147	$3,030	$94	$114	$44

Torres-Rueda S, et al. BMJ Global Health 2022; 6:e005759. doi: 10.1136/bmjgh-2021-005759

2.6 Confirmed cases to date

The table below shows the number of cases confirmed in each country from the start of the pandemic to the end of January 2021, obtained from Dong et al. (2020) (40).

2.6.1 Table SM15: Number of confirmed cases of COVID-19 up to 31 January 2021

Country	Cases reported to 31 January 2021
Afghanistan	55,008
Algeria	107,122
Angola	19,782
Argentina	1,922,264
Bangladesh	534,770
Benin	3786
Bolivia	215,397
Botswana	21,293
Brazil	9,176,975
Burkina Faso	10,580
Burundi	1613
Cabo Verde	13,981
Cambodia	465
Cameroon	29,617
Central African Republic	4981
Chad	3347
Colombia	2,086,806
Comoros	2718
Congo, Dem. Rep.	22,604
Congo, Rep.	7887
Costa Rica	193,276
Cote d'Ivoire	28,178
Dominican Republic	212,553
Ecuador	249,779
Egypt, Arab Rep.	165,418
El Salvador	53,989
Equatorial Guinea	5516
Eswatini	15,666
Ethiopia	137,021
Gabon	10,748
Gambia, The	4090
Ghana	65,427
Guatemala	159,118
Guinea	14,475
Guinea-Bissau	2532
Haiti	11,460
Honduras	147,100
India	10,746,174
Iraq	N/A

Country	Cases reported to 31 January 2021
Jordan	325,674
Kenya	100,675
Lebanon	298,913
Lesotho	8649
Liberia	1939
Libya	117,650
Madagascar	18,743
Malawi	23,497
Mali	8069
Mauritania	16,608
Mauritius	569
Mexico	1,857,230
Morocco	470,691
Mozambique	37,705
Namibia	33,832
Nepal	270,854
Nicaragua	6253
Niger	4516
Nigeria	130,557
Pakistan	544,813
Paraguay	132,548
Peru	1,133,022
Rwanda	15,118
Sao Tome and Principe	1256
Senegal	26,213
Sierra Leone	3528
South Africa	1,449,236
Sri Lanka	63,293
Sudan	29,291
Syrian Arab Republic	13,998
Tajikistan	13,308
Tanzania	509
Togo	5041
Tunisia	207,468
Turkey	2,470,901
Uganda	N/A
West Bank and Gaza	N/A
Yemen, Rep.	2120
Zambia	53,352
Zimbabwe	33,273

Torres-Rueda S, *et al. BMJ Global Health* 2022; 6:e005759. doi: 10.1136/bmjgh-2021-005759

3. References

1. Backer JA, Klinkenberg D, Wallinga J. Incubation period of 2019 novel coronavirus (2019-nCoV) infections among travellers from Wuhan, China, 20-28 January 2020. Euro Surveill. 2020;25(5).
2. Pearson C, van Zandvoort K, Jarvis C, Davies N, Checchi F, CMMID nCov Working Group, et al. Projections of COVID-19 epidemics in LMIC countries. Update: June 2020. 2020 [Available from: https://cmmid.github.io/topics/covid19/LMIC-projection-reports.html.
3. Davies NG, Klepac P, Liu Y, Prem K, Jit M, group CC-w, et al. Age-dependent effects in the transmission and control of COVID-19 epidemics. Nat Med. 2020;26(8):1205-11.
4. Prem K, Cook AR, Jit M. Projecting social contact matrices in 152 countries using contact surveys and demographic data. PLoS Comput Biol. 2017;13(9):e1005697.
5. WorldPop, Center for International Earth Science Information Network, (CIESIN). 2020.
6. Rees EM, Nightingale ES, Jafari Y, Waterlow NR, Clifford S, Pearson CAB, et al. COVID-19 length of hospital stay: a systematic review and data synthesis. medRxiv. 2020:2020.04.30.20084780.
7. Bhatraju PK, Ghassemieh BJ, Nichols M, Kim R, Jerome KR, Nalla AK, et al. Covid-19 in Critically Ill Patients in the Seattle Region - Case Series. N Engl J Med. 2020;382(21):2012-22.
8. Cai Q, Huang D, Ou P, Yu H, Zhu Z, Xia Z, et al. COVID-19 in a designated infectious diseases hospital outside Hubei Province, China. Allergy. 2020;75(7):1742-52.
9. Chen J, Qi T, Liu L, Ling Y, Qian Z, Li T, et al. Clinical progression of patients with COVID-19 in Shanghai, China. J Infect. 2020;80(5):e1-e6.
10. Liu L, Gao J-Y, Hu W-m, Zhang X-x, Guo L, Liu C-q, et al. Clinical characteristics of 51 patients discharged from hospital with COVID-19 in Chongqing,China. medRxiv. 2020:2020.02.20.20025536.
11. Walker PG, Whittaker C, Watson O, Baguelin M, Ainslie K, Bhatia S, et al. The global impact of covid-19 and strategies for mitigation and suppression. WHO Collaborating Centre for Infectious Disease Modelling, MRC Centre for Global Infectious Disease Analysis, Abdul Latif Jameel Institute for Disease and Emergency Analytics, Imperial College London. 2020.
12. Xie H, Zhao J, Lian N, Lin S, Xie Q, Zhuo H. Clinical characteristics of non-ICU hospitalized patients with coronavirus disease 2019 and liver injury: A retrospective study. Liver Int. 2020;40(6):1321-6.
13. Zhao W, Yu S, Zha X, Wang N, Pang Q, Li D, et al. Clinical characteristics and durations of hospitalized patients with COVID-19 in Beijing: a retrospective cohort study. medRxiv. 2020:2020.03.13.20035436.
14. Cao B, Wang Y, Wen D, Liu W, Wang J, Fan G, et al. A Trial of Lopinavir-Ritonavir in Adults Hospitalized with Severe Covid-19. N Engl J Med. 2020;382(19):1787-99.
15. Linton NM, Kobayashi T, Yang Y, Hayashi K, Akhmetzhanov AR, Jung SM, et al. Incubation Period and Other Epidemiological Characteristics of 2019 Novel Coronavirus Infections with Right Truncation: A Statistical Analysis of Publicly Available Case Data. J Clin Med. 2020;9(2).
16. Davies NG, Kucharski AJ, Eggo RM, Gimma A, Edmunds WJ. The effect of non-pharmaceutical interventions on COVID-19 cases, deaths and demand for hospital services in the UK: a modelling study. medRxiv. 2020:2020.04.01.20049908.
17. World Bank. World Bank Open Data. 2020.
18. World Health Organization. COVID-19 Strategic Preparedness and Response Plan: Operational Planning Guidelines to Support Country Preparedness and Response. 2020.
19. Phase DCT. Universal Health Coverage: Essential Package of Health Services for Pakistan: A report for review by the International Advisory Group. Ministry of National Health Services, Government of Pakistan and World Health Organisation,; 2020.
20. Kairu A, Orangi S, Oyando R, Kabia E, Nguhiu P, Ong'ang'o J, et al. The costs of providing TB services in healthcare facilities in Kenya. 51st Union World Conference on Lung Health2020.

21. Chatterjee S, Toshniwal MN, Bhide P, Sachdeva KS, Rao R, Vassall A, et al. Examining the cost of delivering TB prevention, diagnosis and treatment in India, 2018 51st Union World Conference on Lung Health2020.
22. NHSDigital. Hospital admitted patient care activity 2018--19 [Available from: https://digital.nhs.uk/data-andinformation/publications/statistical/hospital-admitted-patient-care-activity/2018-19.
23. Yang X, Yu Y, Xu J, Shu H, Liu H, Wu Y, et al. Clinical course and outcomes of critically ill patients with SARS-CoV-2 pneumonia in Wuhan, China: a single-centered, retrospective, observational study. The Lancet Respiratory Medicine. 2020.
24. Li J, al. e. Epidemiology of COVID-19: A Systematic Review and Meta-analysis of Clinical Characteristics, Risk factors and Outcomes. Journal of Medical Virology.
25. Wang D, Hu B, Hu C, Zhu F, Liu X, Zhang J, et al. Clinical characteristics of 138 hospitalized patients with 2019 novel coronavirus-infected pneumonia in Wuhan, China. JAMA. 2020;323(11):1061-9.
26. Srivastava U. Anaesthesia gas supply: gas cylinders. Indian J Anaesth. 2013;57(5):500-6.
27. Afrox. Medical Oxygen 2020 [Available from: https://www.afroxshop.co.za/shop/en/za/medical-oxygen.
28. Vassall A, Siapka M, Foster N, Cunnama L, Ramma L, Fielding K, et al. Cost-effectiveness of Xpert MTB/RIF for tuberculosis diagnosis in South Africa: a real-world cost analysis and economic evaluation. Lancet Glob Health. 2017;5(7):e710-e9.
29. Khan RM, Albutt K, Qureshi MA, Ansari Z, Drevin G, Mukhopadhyay S, et al. Time-driven activity-based costing of total knee replacements in Karachi, Pakistan. BMJ Open. 2019;9(5):e025258.
30. WHO. COVID-19 Essential Supplies Forecasting Tool. 29 April 2020 [Available from: https://www.who.int/publications/m/item/covid-19-essential-supplies-forecasting-tool#.
31. Serje J, Bertram MY, Brindley C, Lauer JA. Global health worker salary estimates: an econometric analysis of global earnings data. Cost Eff Resour Alloc. 2018;16:10.
32. World Health Organization. Global Health Observatory (GHO) data [Available from: http://www.who.int/gho/en/.
33. Nishiura H, Linton NM, Akhmetzhanov AR. Serial interval of novel coronavirus (COVID-19) infections. medRxiv. 2020:2020.02.03.20019497.
34. Health KMo. COVID-19 Outbreak in Kenya. Daily Situation Report -78 [updated 3 June 2020. Available from: https://www.health.go.ke/wp-content/uploads/2020/06/Kenya-COVID-19-SITREP-078-03-Jun-2020.pdf.
35. Africa RoS. COVID-19 Corona Virus South African Resource Porta 2020 [Available from: https://sacoronavirus.co.za/.
36. Ethiopia MoHo. Ethiopia COVID 19 Monitoring Platform 2020 [Available from: http://www.moh.gov.et/ejcc/en/node/196.
37. India Go. COVID Statewide Status [Available from: https://www.mygov.in/corona-data/covid19-statewise-status/.
38. Pakistan Go. COVID19 Health Advisory Platform [Available from: http://covid.gov.pk/.
39. Organization WH. WHO Global Health Expenditure Database.
40. Dong E, Du H, Gardner L. An interactive web-based dashboard to track COVID-19 in real time. Lancet Infect Dis. 2020;20(5):533-4.
