# Supplementary material for: Stark choices: exploring health sector costs of policy responses to COVID-19 in low-income and middle-income countries
Source: BMJ Glob Health. 2021 Dec 2;6(12):e005759. doi: 10.1136/bmjgh-2021-005759 (PMC8640196; doi:10.1136/bmjgh-2021-005759)
Supplement: Supplementary data [file bmjgh-2021-005759supp002.pdf]

Supplementary Results Appendix

Supplemental Results Tables

Table SR1: Unit Costs per Activity per Country

Country	1.a. Emergency Response Mechanisms: National level (per country per day)	1.b. Emergency Response Mechanisms: Training of health staff (one-off per site)	2. Risk communication & community engagement (per country per day)	3.a. Case finding, contact tracing and management: Contact tracing (per person contacted)	3.b. Case finding, contact tracing and management: Quarantine of contacts (per person quarantined)	4.a. Surveillance: Case notification (per positive case)	4.b. Surveillance: Reporting (national level) (per country per week)	5. Public health measures: Hygiene education (per education campaign per month)	6. Screening and diagnosis (per person screened and tested)	7.a. Case Management: Home-based care (per person requiring home-based care)	7.b. Case Management: Hospital-based (severe case) (per day of hospitalisation (severe case)	7.c. Case Management: Hospital-based (critical case) (per day of hospitalisation (critical case)	7.d. Case Management: Death (per COVID-related death)
Afghanistan	$946.00	$6,705.01	$82.56	$2.29	$2.36	$2.36	$5.96	$49.97	$29.40	$14.55	$33.80	$262.79	$64.52
Albania	$3,789.37	$27,990.77	$324.45	$12.58	$9.38	$9.38	$24.29	$196.86	$51.28	$142.99	$104.61	$1,115.20	$64.52
Algeria	$3,295.01	$24,748.13	$281.48	$12.21	$8.19	$8.19	$21.48	$171.15	$52.63	$155.05	$111.21	$1,184.99	$64.52
Angola	$3,912.28	$27,196.03	$345.35	$8.64	$9.82	$9.82	$24.60	$208.91	$35.90	$43.99	$45.96	$322.04	$64.52
Argentina	$7,558.99	$53,724.40	$669.23	$22.98	$19.26	$19.26	$49.36	$405.38	$71.04	$238.55	$155.69	$1,539.72	$64.52
Armenia	$3,070.21	$23,010.80	$257.48	$10.06	$7.44	$7.44	$19.25	$156.20	$45.41	$111.63	$87.77	$967.20	$64.52
Azerbaijan	$3,752.25	$27,941.99	$324.53	$14.02	$9.45	$9.45	$24.79	$197.35	$57.18	$180.13	$124.70	$1,305.33	$64.52
Bangladesh	$2,059.69	$14,725.23	$177.44	$4.64	$5.06	$5.06	$12.76	$107.44	$30.00	$24.18	$35.87	$256.34	$64.52
Belarus	$4,683.68	$34,307.35	$409.84	$16.64	$11.89	$11.89	$31.01	$248.96	$62.27	$205.02	$138.01	$1,416.77	$64.52
Belize	$3,333.58	$24,713.90	$279.96	$10.07	$8.06	$8.06	$20.65	$169.59	$43.97	$100.66	$81.79	$906.83	$64.52
Benin	$1,553.93	$10,800.31	$137.55	$3.65	$3.92	$3.92	$9.84	$83.18	$31.95	$21.93	$38.15	$306.00	$64.52
Bhutan	$3,922.04	$27,434.10	$348.99	$8.79	$9.97	$9.97	$25.27	$211.45	$37.31	$46.65	$46.72	$326.98	$64.52
Bolivia	$4,105.27	$28,551.20	$363.74	$9.10	$10.36	$10.36	$26.03	$220.13	$36.96	$46.88	$47.24	$330.38	$64.52
Bosnia and Herzegovina	$4,276.91	$31,332.89	$369.28	$14.03	$10.67	$10.67	$27.58	$224.00	$54.25	$157.93	$112.61	$1,183.29	$64.52
Botswana	$5,646.94	$40,743.98	$495.58	$18.25	$14.31	$14.31	$36.91	$300.51	$63.18	$203.55	$137.06	$1,393.13	$64.52
Brazil	$5,860.45	$42,075.40	$512.96	$17.88	$14.77	$14.77	$37.87	$310.75	$60.54	$185.49	$127.26	$1,297.35	$64.52
Bulgaria	$6,360.94	$45,703.09	$562.32	$20.86	$16.24	$16.24	$41.97	$341.06	$69.45	$237.46	$155.28	$1,554.18	$64.52
Burkina Faso	$1,244.93	$8,709.59	$109.45	$2.93	$3.12	$3.12	$7.84	$66.19	$30.28	$17.60	$35.44	$274.40	$64.52
Burundi	$499.56	$3,658.28	$41.54	$1.16	$1.18	$1.18	$2.97	$25.12	$25.93	$6.65	$28.43	$189.56	$64.52

Country	1.a. Emergency Response Mechanisms: National level (per country per day)	1.b. Emergency Response Mechanisms: Training of health staff (one-off per site)	2. Risk communication & community engagement (per country per day)	3.a. Case finding, contact tracing and management: Contact tracing (per person contacted)	3.b. Case finding, contact tracing and management: Quarantine of contacts (per person quarantined)	4.a. Surveillance: Case notification (per positive case)	4.b. Surveillance: Reporting (national level) (per country per week)	5. Public health measures: Hygiene education (per education campaign per month)	6. Screening and diagnosis (per person screened and tested)	7.a. Case Management: Home-based care (per person requiring home-based care)	7.b. Case Management: Hospital-based (severe case) (per day of hospitalisation (severe case)	7.c. Case Management: Hospital-based (critical case) (per day of hospitalisation (critical case)	7.d. Case Management: Death (per COVID-related death)
Cabo Verde	$4,169.90	$28,963.40	$369.23	$9.22	$10.51	$10.51	$26.36	$223.41	$36.98	$47.23	$47.50	$332.06	$64.52
Cambodia	$1,870.45	$13,460.69	$160.44	$4.24	$4.58	$4.58	$11.58	$97.18	$29.47	$22.30	$34.88	$249.88	$64.52
Cameroon	$1,863.02	$13,384.27	$159.34	$4.21	$4.54	$4.54	$11.44	$96.46	$29.23	$21.83	$34.73	$248.91	$64.52
Central African Republic	$818.01	$5,797.62	$70.25	$1.85	$2.00	$2.00	$4.98	$42.44	$27.02	$10.15	$30.39	$205.95	$64.52
Chad	$1,264.27	$8,838.47	$111.18	$2.97	$3.17	$3.17	$7.96	$67.23	$30.31	$17.75	$35.50	$274.26	$64.52
China	$6,423.38	$45,963.82	$565.22	$19.78	$16.28	$16.28	$41.78	$342.46	$64.84	$208.24	$139.47	$1,404.02	$64.52
Colombia	$4,615.10	$33,630.30	$400.02	$14.87	$11.55	$11.55	$29.79	$242.57	$55.69	$164.42	$116.07	$1,210.70	$64.52
Comoros	$1,697.25	$12,236.47	$143.81	$3.83	$4.09	$4.09	$10.27	$87.01	$28.45	$19.58	$33.70	$242.18	$64.52
Congo, Dem. Rep.	$953.63	$6,709.71	$82.49	$2.15	$2.34	$2.34	$5.84	$49.83	$27.53	$11.70	$31.28	$214.03	$64.52
Congo, Rep.	$2,580.26	$18,262.28	$225.15	$5.79	$6.42	$6.42	$16.20	$136.33	$31.92	$30.24	$38.84	$275.67	$64.52
Costa Rica	$7,582.00	$53,753.01	$669.15	$22.07	$19.22	$19.22	$49.05	$405.06	$67.34	$215.23	$143.07	$1,420.29	$64.52
Cote d'Ivoire	$2,068.18	$14,774.26	$178.08	$4.66	$5.08	$5.08	$12.78	$107.81	$29.96	$24.15	$35.89	$256.42	$64.52
Dominica	$4,945.79	$35,693.21	$426.96	$14.31	$12.26	$12.26	$31.28	$258.44	$51.63	$136.59	$100.96	$1,063.14	$64.52
Dominican Republic	$5,490.71	$39,653.09	$480.87	$17.63	$13.88	$13.88	$35.78	$291.57	$61.63	$195.05	$132.49	$1,352.33	$64.52
Ecuador	$4,268.78	$31,153.65	$366.44	$13.07	$10.55	$10.55	$27.08	$222.01	$50.53	$134.70	$100.05	$1,064.85	$64.52
Egypt, Arab Rep.	$3,345.86	$23,690.07	$298.93	$7.65	$8.60	$8.60	$22.07	$181.46	$36.55	$42.49	$44.13	$310.14	$64.52
El Salvador	$4,641.54	$32,154.42	$412.24	$10.25	$11.73	$11.73	$29.44	$249.43	$38.61	$52.51	$50.13	$349.22	$64.52
Equatorial Guinea	$6,924.23	$49,535.56	$613.62	$22.32	$17.71	$17.71	$45.67	$372.06	$72.01	$249.34	$161.62	$1,605.38	$64.52
Eswatini	$4,853.50	$33,677.50	$432.99	$10.78	$12.35	$12.35	$31.13	$262.16	$40.06	$56.21	$51.68	$359.30	$64.52
Ethiopia	$804.71	$4,813.58	$74.14	$3.48	$1.72	$1.72	$3.69	$44.58	$36.97	$22.90	$35.29	$505.56	$64.52

Country	1.a. Emergency Response Mechanisms: National level (per country per day)	1.b. Emergency Response Mechanisms: Training of health staff (one-off per site)	2. Risk communication & community engagement (per country per day)	3.a. Case finding, contact tracing and management: Contact tracing (per person contacted)	3.b. Case finding, contact tracing and management: Quarantine of contacts (per person quarantined)	4.a. Surveillance: Case notification (per positive case)	4.b. Surveillance: Reporting (national level) (per country per week)	5. Public health measures: Hygiene education (per education campaign per month)	6. Screening and diagnosis (per person screened and tested)	7.a. Case Management: Home-based care (per person requiring home-based care)	7.b. Case Management: Hospital-based (severe case) (per day of hospitalisation (severe case)	7.c. Case Management: Hospital-based (critical case) (per day of hospitalisation (critical case)	7.d. Case Management: Death (per COVID-related death)
Fiji	$4,181.80	$30,530.23	$357.98	$12.60	$10.30	$10.30	$26.39	$216.83	$49.19	$126.98	$95.89	$1,026.93	$64.52
Gabon	$5,445.87	$39,356.19	$476.93	$17.58	$13.77	$13.77	$35.51	$289.20	$61.67	$195.62	$132.81	$1,356.03	$64.52
Gambia, The	$1,280.15	$8,974.04	$113.08	$3.10	$3.23	$3.23	$8.16	$68.44	$31.54	$19.75	$37.19	$305.66	$64.52
Georgia	$3,425.61	$25,479.11	$290.70	$11.36	$8.41	$8.41	$21.76	$176.38	$48.53	$128.48	$96.82	$1,047.23	$64.52
Ghana	$2,585.83	$18,256.88	$224.96	$5.77	$6.40	$6.40	$16.09	$136.14	$31.59	$29.66	$38.70	$274.73	$64.52
Grenada	$6,737.67	$47,967.95	$591.57	$19.58	$16.99	$16.99	$43.35	$358.09	$62.28	$189.89	$129.50	$1,305.14	$64.52
Guatemala	$3,146.69	$23,445.60	$262.99	$9.61	$7.57	$7.57	$19.44	$159.34	$43.21	$97.32	$80.01	$892.89	$64.52
Guinea	$1,523.11	$10,597.42	$134.84	$3.60	$3.84	$3.84	$9.66	$81.55	$32.02	$21.85	$38.19	$308.79	$64.52
Guinea-Bissau	$1,331.15	$9,278.97	$117.07	$3.09	$3.33	$3.33	$8.35	$70.77	$30.18	$17.93	$35.42	$268.43	$64.52
Guyana	$3,382.85	$25,045.87	$284.39	$10.17	$8.18	$8.18	$20.96	$172.26	$44.10	$101.10	$82.02	$908.21	$64.52
Haiti	$1,473.11	$10,233.43	$129.88	$3.40	$3.69	$3.69	$9.25	$78.50	$30.70	$19.53	$36.33	$276.50	$64.52
Honduras	$2,905.84	$20,412.59	$253.99	$6.46	$7.23	$7.23	$18.14	$153.68	$32.62	$33.10	$40.44	$286.12	$64.52
India	$2,488.27	$14,521.07	$223.48	$13.97	$5.45	$5.45	$11.11	$135.95	$42.99	$72.83	$44.68	$377.77	$64.52
Indonesia	$4,731.35	$32,975.24	$423.84	$10.61	$12.12	$12.12	$30.78	$256.87	$40.63	$56.68	$51.50	$358.17	$64.52
Iraq	$4,306.03	$31,650.34	$373.96	$15.00	$10.84	$10.84	$28.21	$227.08	$57.94	$180.76	$124.95	$1,299.28	$64.52
Jamaica	$3,617.43	$26,657.34	$306.02	$10.89	$8.81	$8.81	$22.56	$185.36	$45.63	$108.93	$86.22	$944.24	$64.52
Jordan	$3,039.43	$22,766.02	$254.08	$9.71	$7.33	$7.33	$18.92	$154.06	$44.22	$104.40	$83.86	$930.81	$64.52
Kazakhstan	$6,963.51	$50,021.67	$620.92	$24.07	$17.99	$17.99	$46.72	$376.95	$78.71	$290.89	$184.08	$1,816.75	$64.52
Kenya	$3,729.58	$19,756.31	$374.01	$9.10	$6.40	$6.40	$12.22	$218.82	$45.69	$46.08	$45.20	$417.83	$64.52
Kiribati	$1,874.79	$13,393.53	$159.29	$4.18	$4.52	$4.52	$11.27	$96.29	$28.71	$20.92	$34.51	$247.46	$64.52
Kosovo	$3,171.75	$23,738.86	$267.36	$10.60	$7.74	$7.74	$20.05	$162.25	$46.99	$120.70	$92.65	$1,011.76	$64.52

Country	1.a. Emergency Response Mechanisms: National level (per country per day)	1.b. Emergency Response Mechanisms: Training of health staff (one-off per site)	2. Risk communication & community engagement (per country per day)	3.a. Case finding, contact tracing and management: Contact tracing (per person contacted)	3.b. Case finding, contact tracing and management: Quarantine of contacts (per person quarantined)	4.a. Surveillance: Case notification (per positive case)	4.b. Surveillance: Reporting (national level) (per country per week)	5. Public health measures: Hygiene education (per education campaign per month)	6. Screening and diagnosis (per person screened and tested)	7.a. Case Management: Home-based care (per person requiring home-based care)	7.b. Case Management: Hospital-based (severe case) (per day of hospitalisation (severe case)	7.c. Case Management: Hospital-based (critical case) (per day of hospitalisation (critical case)	7.d. Case Management: Death (per COVID-related death)
Kyrgyz Republic	$1,615.02	$11,732.46	$137.15	$3.69	$3.92	$3.92	$9.92	$83.09	$28.58	$19.44	$33.45	$240.58	$64.52
Lao PDR	$3,072.00	$21,629.50	$270.63	$6.90	$7.73	$7.73	$19.55	$163.93	$33.94	$36.34	$41.75	$294.63	$64.52
Lebanon	$5,355.43	$38,539.74	$465.28	$15.82	$13.37	$13.37	$34.19	$281.72	$55.27	$156.30	$111.56	$1,156.87	$64.52
Lesotho	$1,597.13	$11,582.04	$135.04	$3.62	$3.85	$3.85	$9.70	$81.76	$28.29	$18.80	$33.23	$239.15	$64.52
Liberia	$1,150.73	$8,047.64	$100.49	$2.63	$2.86	$2.86	$7.14	$60.72	$28.77	$14.73	$33.25	$238.78	$64.52
Libya	$5,226.52	$38,001.32	$459.29	$18.05	$13.31	$13.31	$34.58	$278.84	$64.76	$216.59	$144.18	$1,466.69	$64.52
Madagascar	$952.69	$6,746.95	$83.12	$2.30	$2.37	$2.37	$5.99	$50.30	$29.30	$14.43	$33.68	$259.97	$64.52
Malawi	$710.11	$5,094.95	$60.88	$1.69	$1.74	$1.74	$4.38	$36.84	$27.56	$10.37	$30.95	$223.90	$64.52
Malaysia	$7,995.53	$57,149.41	$716.71	$27.55	$20.75	$20.75	$53.89	$435.06	$86.58	$332.42	$206.38	$2,011.43	$64.52
Maldives	$6,560.96	$46,765.52	$575.47	$19.12	$16.53	$16.53	$42.19	$348.36	$61.47	$186.13	$127.50	$1,288.92	$64.52
Mali	$1,545.24	$10,737.16	$136.69	$3.62	$3.89	$3.89	$9.77	$82.65	$31.73	$21.53	$37.84	$300.53	$64.52
Marshall Islands	$2,505.43	$18,928.40	$201.97	$6.78	$5.78	$5.78	$14.66	$122.13	$35.70	$55.09	$57.28	$688.14	$64.52
Mauritania	$1,536.44	$11,220.00	$130.29	$3.53	$3.73	$3.73	$9.48	$78.97	$28.47	$18.84	$33.09	$238.23	$64.52
Mauritius	$7,493.11	$53,415.36	$665.59	$23.86	$19.20	$19.20	$49.44	$403.49	$74.89	$263.07	$168.95	$1,665.91	$64.52
Mexico	$6,458.46	$46,269.62	$569.56	$20.38	$16.42	$16.42	$42.25	$345.23	$67.00	$221.47	$146.62	$1,470.97	$64.52
Micronesia, Fed. Sts.	$3,892.92	$26,931.86	$341.45	$8.50	$9.67	$9.67	$24.00	$206.29	$34.76	$41.79	$45.30	$317.78	$64.52
Moldova	$3,750.44	$26,153.16	$331.43	$8.33	$9.44	$9.44	$23.73	$200.59	$35.75	$42.95	$45.27	$317.54	$64.52
Mongolia	$4,999.07	$34,796.91	$448.42	$11.20	$12.83	$12.83	$32.56	$271.76	$41.64	$59.84	$53.04	$368.19	$64.52
Montenegro	$6,066.95	$43,665.79	$534.92	$19.82	$15.45	$15.45	$39.90	$324.42	$67.00	$224.35	$148.24	$1,492.23	$64.52
Morocco	$3,831.70	$26,757.16	$339.70	$8.55	$9.69	$9.69	$24.45	$205.70	$36.47	$44.66	$45.94	$321.94	$64.52
Mozambique	$885.81	$6,279.14	$76.79	$2.09	$2.19	$2.19	$5.51	$46.44	$28.32	$12.54	$32.24	$237.00	$64.52

Country	1.a. Emergency Response Mechanisms: National level (per country per day)	1.b. Emergency Response Mechanisms: Training of health staff (one-off per site)	2. Risk communication & community engagement (per country per day)	3.a. Case finding, contact tracing and management: Contact tracing (per person contacted)	3.b. Case finding, contact tracing and management: Quarantine of contacts (per person quarantined)	4.a. Surveillance: Case notification (per positive case)	4.b. Surveillance: Reporting (national level) (per country per week)	5. Public health measures: Hygiene education (per education campaign per month)	6. Screening and diagnosis (per person screened and tested)	7.a. Case Management: Home-based care (per person requiring home-based care)	7.b. Case Management: Hospital-based (severe case) (per day of hospitalisation (severe case)	7.c. Case Management: Hospital-based (critical case) (per day of hospitalisation (critical case)	7.d. Case Management: Death (per COVID-related death)
Myanmar	$1,809.65	$13,162.27	$156.70	$4.19	$4.51	$4.51	$11.59	$95.14	$30.16	$23.30	$35.00	$250.68	$64.52
Namibia	$4,016.96	$29,428.13	$343.30	$12.32	$9.89	$9.89	$25.38	$208.00	$49.02	$127.13	$95.99	$1,030.42	$64.52
Nauru	$6,392.50	$45,680.97	$561.18	$19.15	$16.14	$16.14	$41.31	$339.86	$62.54	$194.05	$131.80	$1,332.12	$64.52
Nepal	$1,792.27	$12,427.73	$159.47	$4.26	$4.55	$4.55	$11.44	$96.46	$33.84	$26.20	$41.06	$345.98	$64.52
Nicaragua	$2,454.02	$17,413.13	$213.71	$5.52	$6.10	$6.10	$15.40	$129.42	$31.52	$28.90	$38.16	$271.21	$64.52
Niger	$734.43	$5,246.75	$62.89	$1.71	$1.79	$1.79	$4.49	$38.02	$27.17	$9.90	$30.46	$212.86	$64.52
Nigeria	$2,477.88	$17,593.74	$216.20	$5.59	$6.17	$6.17	$15.62	$130.96	$31.76	$29.45	$38.37	$272.59	$64.52
North Macedonia	$4,379.64	$32,100.40	$379.80	$14.81	$10.99	$10.99	$28.51	$230.50	$56.76	$172.86	$120.67	$1,257.73	$64.52
Pakistan	$1,040.21	$8,096.53	$91.67	$2.54	$2.35	$2.35	$6.52	$54.66	$26.98	$12.45	$33.32	$221.18	$64.52
Papua New Guinea	$3,093.77	$21,630.47	$270.27	$6.84	$7.67	$7.67	$19.17	$163.43	$32.84	$34.41	$41.27	$291.52	$64.52
Paraguay	$4,093.41	$30,051.30	$352.01	$13.30	$10.17	$10.17	$26.26	$213.49	$52.43	$147.91	$107.22	$1,135.18	$64.52
Peru	$4,727.22	$34,360.81	$409.68	$14.92	$11.81	$11.81	$30.41	$248.33	$55.24	$160.77	$114.07	$1,190.19	$64.52
Philippines	$3,715.60	$25,998.60	$329.55	$8.31	$9.41	$9.41	$23.79	$199.61	$36.28	$43.76	$45.41	$318.44	$64.52
Romania	$8,292.36	$58,997.17	$740.82	$27.01	$21.39	$21.39	$55.21	$449.26	$82.82	$306.72	$192.43	$1,875.29	$64.52
Russian Federation	$7,702.04	$54,970.61	$686.89	$25.41	$19.85	$19.85	$51.30	$416.65	$79.82	$292.36	$184.76	$1,811.91	$64.52
Rwanda	$1,347.77	$9,410.86	$118.90	$3.19	$3.39	$3.39	$8.52	$71.91	$31.06	$19.39	$36.64	$290.68	$64.52
Samoa	$2,847.73	$21,344.19	$234.62	$8.33	$6.74	$6.74	$17.23	$142.05	$39.85	$78.49	$69.88	$801.79	$64.52
Sao Tome and Principe	$2,312.52	$16,369.60	$199.43	$5.15	$5.67	$5.67	$14.17	$120.61	$30.34	$26.03	$37.01	$263.77	$64.52
Senegal	$1,851.05	$13,304.20	$158.26	$4.18	$4.51	$4.51	$11.37	$95.81	$29.20	$21.71	$34.67	$248.50	$64.52
Serbia	$5,050.29	$36,669.46	$440.98	$16.59	$12.74	$12.74	$32.93	$267.49	$60.00	$188.16	$128.83	$1,324.55	$64.52
Sierra Leone	$947.08	$6,696.22	$82.40	$2.24	$2.35	$2.35	$5.91	$49.84	$28.75	$13.55	$32.92	$245.91	$64.52

Country	1.a. Emergency Response Mechanisms: National level (per country per day)	1.b. Emergency Response Mechanisms: Training of health staff (one-off per site)	2. Risk communication & community engagement (per country per day)	3.a. Case finding, contact tracing and management: Contact tracing (per person contacted)	3.b. Case finding, contact tracing and management: Quarantine of contacts (per person quarantined)	4.a. Surveillance: Case notification (per positive case)	4.b. Surveillance: Reporting (national level) (per country per week)	5. Public health measures: Hygiene education (per education campaign per month)	6. Screening and diagnosis (per person screened and tested)	7.a. Case Management: Home-based care (per person requiring home-based care)	7.b. Case Management: Hospital-based (severe case) (per day of hospitalisation (severe case)	7.c. Case Management: Hospital-based (critical case) (per day of hospitalisation (critical case)	7.d. Case Management: Death (per COVID-related death)
Solomon Islands	$2,396.09	$16,881.46	$206.20	$5.29	$5.84	$5.84	$14.53	$124.60	$30.20	$26.17	$37.26	$265.38	$64.52
South Africa	$11,590.43	$68,141.36	$1,133.44	$26.23	$29.22	$29.22	$68.26	$682.05	$73.12	$146.57	$105.88	$1,081.94	$64.52
Sri Lanka	$3,180.67	$23,890.53	$269.72	$11.31	$7.83	$7.83	$20.44	$163.87	$49.74	$137.82	$101.91	$1,098.98	$64.52
St. Lucia	$6,608.38	$47,023.86	$578.70	$18.76	$16.60	$16.60	$42.27	$350.17	$59.77	$175.17	$121.56	$1,232.20	$64.52
St. Vincent and the Grenadines	$4,834.16	$34,990.28	$417.76	$14.45	$12.01	$12.01	$30.75	$253.00	$52.80	$144.76	$105.40	$1,106.71	$64.52
Sudan	$1,356.75	$10,048.11	$114.60	$3.17	$3.30	$3.30	$8.46	$69.57	$28.20	$17.48	$32.27	$232.87	$64.52
Suriname	$4,413.28	$32,290.71	$382.21	$14.61	$11.05	$11.05	$28.59	$231.88	$55.77	$166.40	$117.17	$1,224.19	$64.52
Syrian Arab Republic	$3,155.07	$21,420.41	$279.68	$6.69	$7.90	$7.90	$19.47	$168.67	$31.91	$30.88	$40.45	$244.31	$64.52
Tajikistan	$1,494.31	$10,447.28	$132.96	$3.68	$3.80	$3.80	$9.63	$80.50	$33.68	$24.27	$40.41	$353.10	$64.52
Tanzania	$1,825.32	$12,655.42	$162.54	$4.35	$4.63	$4.63	$11.67	$98.33	$34.19	$26.92	$41.58	$353.69	$64.52
Thailand	$5,151.28	$37,420.12	$451.26	$17.33	$13.05	$13.05	$33.83	$273.84	$62.36	$202.11	$136.36	$1,394.09	$64.52
Timor-Leste	$2,574.86	$18,319.10	$226.15	$5.85	$6.48	$6.48	$16.50	$137.12	$32.65	$31.56	$39.19	$277.97	$64.52
Togo	$1,178.69	$8,256.12	$103.34	$2.76	$2.94	$2.94	$7.39	$62.49	$29.71	$16.34	$34.56	$262.03	$64.52
Tonga	$2,939.45	$21,960.04	$242.84	$8.50	$6.97	$6.97	$17.80	$147.00	$40.02	$78.89	$70.08	$802.30	$64.52
Tunisia	$4,252.55	$29,750.38	$380.41	$9.57	$10.89	$10.89	$27.71	$230.62	$39.09	$51.54	$48.89	$341.14	$64.52
Turkey	$6,740.55	$48,526.20	$600.98	$23.66	$17.42	$17.42	$45.34	$364.94	$78.33	$290.19	$183.74	$1,816.90	$64.52
Turkmenistan	$5,003.13	$36,431.94	$438.10	$17.10	$12.69	$12.69	$32.93	$265.93	$62.27	$202.67	$136.69	$1,399.39	$64.52
Tuvalu	$2,462.60	$18,642.18	$198.16	$6.71	$5.68	$5.68	$14.40	$119.84	$35.66	$55.15	$57.32	$689.16	$64.52
Uganda	$1,137.04	$7,988.99	$99.80	$2.71	$2.85	$2.85	$7.17	$60.37	$30.08	$16.68	$35.01	$273.28	$64.52
Ukraine	$3,723.18	$26,062.40	$330.44	$8.34	$9.44	$9.44	$23.88	$200.18	$36.41	$44.03	$45.50	$319.05	$64.52
Uzbekistan	$2,117.99	$15,309.31	$185.79	$4.91	$5.36	$5.36	$13.81	$112.86	$31.72	$27.65	$36.98	$263.52	$64.52

BMJ Publishing Group Limited (BMJ) disclaims all liability and responsibility arising from any reliance placed on this supplemental material which has been supplied by the author(s)

Country	1.a. Emergency Response Mechanisms: National level (per country per day)	1.b. Emergency Response Mechanisms: Training of health staff (one-off per site)	2. Risk communication & community engagement (per country per day)	3.a. Case finding, contact tracing and management: Contact tracing (per person contacted)	3.b. Case finding, contact tracing and management: Quarantine of contacts (per person quarantined)	4.a. Surveillance: Case notification (per positive case)	4.b. Surveillance: Reporting (national level) (per country per week)	5. Public health measures: Hygiene education (per education campaign per month)	6. Screening and diagnosis (per person screened and tested)	7.a. Case Management: Home-based care (per person requiring home-based care)	7.b. Case Management: Hospital-based (severe case) (per day of hospitalisation (severe case)	7.c. Case Management: Hospital-based (critical case) (per day of hospitalisation (critical case)	7.d. Case Management: Death (per COVID-related death)
Vanuatu	$3,428.98	$23,817.61	$299.54	$7.51	$8.48	$8.48	$21.07	$180.98	$33.36	$36.96	$42.82	$301.56	$64.52
Vietnam	$3,096.65	$21,794.49	$272.85	$6.95	$7.79	$7.79	$19.70	$165.27	$34.01	$36.59	$41.88	$295.48	$64.52
West Bank and Gaza	$3,608.29	$25,105.36	$317.09	$7.95	$9.00	$9.00	$22.50	$191.74	$34.57	$40.08	$44.12	$310.07	$64.52
Yemen, Rep.	$1,627.62	$11,301.00	$144.29	$3.83	$4.11	$4.11	$10.33	$87.26	$32.44	$23.09	$38.92	$315.72	$64.52
Zambia	$1,892.78	$13,60[illegible].47	$162.34	$4.29	$4.63	$4.63	$11.70	$98.31	$29.48	$22.43	$34.97	$250.47	$64.52
Zimbabwe	$2,438.05	$17,1[illegible].94	$210.42	$5.40	$5.97	$5.97	$14.89	$127.20	$30.54	$27.01	$37.60	$267.56	$64.52

Table SR2. Total Annual Costs per Country per Scenario (2019 US$)

Country	Scenario 1: No mitigation	Scenario 2: Contact reduction: high symptomatic cases/low general population	Scenario 3: Contact reduction: high symptomatic cases/high general population	Scenario 4: 30-day lockdown + low contact reduction general population
Afghanistan	$1,311,815,462	$1,177,904,724	$25,590,730	$1,399,789,895
Algeria	$6,603,396,550	$4,797,609,340	$108,875,236	$6,084,882,367
Angola	$1,390,745,332	$1,335,741,419	$32,119,637	$1,567,094,020
Argentina	$15,973,976,990	$12,132,966,200	$297,934,143	$15,001,801,214
Bangladesh	$8,389,029,878	$7,346,626,409	$145,098,719	$8,762,131,868
Benin	$507,979,013	$463,323,321	$9,006,214	$544,803,973
Bolivia	$711,164,156	$607,984,156	$12,278,798	$738,627,623
Botswana	$380,200,596	$303,116,161	$8,450,322	$369,522,644
Brazil	$53,346,806,690	$40,013,304,222	$804,437,570	$49,832,168,384
Burkina Faso	$775,230,046	$719,748,836	$15,228,297	$840,119,232
Burundi	$330,390,715	$304,544,873	$6,022,985	$359,734,439
Cabo Verde	$32,485,358	$28,162,920	$1,977,680	$34,414,567
Cambodia	$678,417,754	$566,274,013	$10,441,999	$690,985,182
Cameroon	$949,939,675	$884,467,637	$18,998,192	$1,043,034,826
Central African Republic	$149,319,610	$137,035,951	$3,386,607	$161,434,468
Chad	$537,334,959	$471,303,516	$10,468,642	$571,410,658
Colombia	$11,249,962,883	$8,475,322,329	$160,659,652	$10,533,617,075
Comoros	$32,394,068	$29,677,244	$1,121,321	$35,281,984
Congo, Dem. Rep.	$2,841,214,584	$2,605,619,705	$46,150,170	$3,067,476,753
Congo, Rep.	$227,099,135	$211,912,215	$6,037,737	$249,919,561
Costa Rica	$1,400,074,702	$1,042,161,571	$22,704,715	$1,305,750,553
Cote d'Ivoire	$986,545,276	$890,204,132	$15,185,093	$1,059,989,337
Dominican Republic	$2,373,818,269	$1,828,777,716	$38,858,617	$2,256,950,992
Ecuador	$3,039,528,826	$2,380,985,925	$47,610,269	$2,908,422,164
Egypt, Arab Rep.	$5,473,593,518	$4,577,530,923	$111,791,634	$5,670,659,187
El Salvador	$518,955,578	$434,268,407	$10,846,582	$530,457,154
Equatorial Guinea	$201,292,014	$178,959,540	$5,834,851	$210,653,340
Eswatini	$61,060,003	$56,029,505	$2,763,892	$67,309,663
Ethiopia	$6,504,466,772	$5,627,795,574	$106,922,008	$6,683,485,989
Gabon	$317,521,713	$268,464,549	$7,663,635	$320,961,919
Gambia, The	$99,167,588	$90,763,358	$2,372,778	$106,107,646
Ghana	$1,357,588,372	$1,221,891,384	$21,852,775	$1,453,846,283
Guatemala	$1,837,045,148	$1,483,251,542	$31,968,720	$1,795,402,698
Guinea	$563,640,662	$509,527,263	$10,664,897	$600,953,264
Guinea-Bissau	$75,923,827	$70,013,569	$1,851,100	$82,337,549
Haiti	$555,317,592	$482,130,225	$9,866,715	$577,852,090
Honduras	$501,268,135	$449,603,344	$10,781,520	$534,058,049
India	$109,620,866,995	$94,845,714,832	$2,103,684,598	$113,697,199,394
Iraq	$5,183,672,685	$4,325,177,524	$90,063,401	$5,178,286,305
Jordan	$927,233,188	$747,309,105	$20,519,181	$908,958,207

Kenya	$3,484,374,138	$3,372,960,785	$80,811,173	$3,829,322,849
Lebanon	$1,487,047,117	$1,169,981,518	$33,200,071	$1,424,189,709
Lesotho	$86,850,141	$73,935,288	$1,834,725	$89,815,951
Liberia	$191,603,380	$174,077,339	$4,107,994	$205,216,814
Libya	$1,193,918,874	$916,036,535	$24,085,390	$1,140,287,493
Madagascar	$1,047,585,263	$929,396,816	$17,329,515	$1,108,391,195
Malawi	$607,876,121	$549,184,096	$10,742,706	$651,787,586
Mali	$710,566,778	$622,492,840	$13,497,485	$755,421,554
Mauritania	$171,731,082	$157,899,063	$4,306,691	$186,164,959
Mauritius	$504,657,186	$370,884,267	$10,115,829	$465,076,712
Mexico	$31,608,715,789	$24,625,827,163	$553,234,603	$30,216,622,750
Morocco	$2,875,202,949	$2,431,152,726	$57,666,385	$2,940,380,785
Mozambique	$1,044,905,442	$940,866,088	$18,016,630	$1,119,434,522
Namibia	$230,201,450	$195,667,209	$5,412,342	$233,404,625
Nepal	$1,794,390,666	$1,521,796,884	$26,163,458	$1,827,252,564
Nicaragua	$327,475,896	$285,335,078	$6,578,531	$342,217,217
Niger	$682,188,023	$599,341,108	$12,782,840	$725,945,647
Nigeria	$8,523,755,754	$7,962,255,548	$163,489,812	$9,326,514,000
Pakistan	$7,911,623,290	$6,868,761,344	$152,483,404	$8,242,469,426
Paraguay	$1,105,713,068	$850,884,034	$16,848,189	$1,053,013,278
Peru	$6,627,248,434	$4,985,660,537	$89,939,295	$6,187,688,798
Rwanda	$522,348,774	$471,398,249	$9,991,356	$558,014,561
Sao Tome and Principe	$8,558,390	$8,669,252	$863,312	$10,042,513
Senegal	$647,876,841	$595,256,052	$13,416,866	$699,179,799
Sierra Leone	$294,071,444	$268,216,325	$5,784,691	$316,044,465
South Africa	$9,845,785,451	$8,334,446,288	$220,526,183	$10,104,268,308
Sri Lanka	$5,352,138,415	$4,065,040,082	$94,898,307	$4,988,596,480
Sudan	$1,622,278,153	$1,465,299,051	$29,202,119	$1,732,370,209
Syrian Arab Republic	$724,943,647	$683,467,982	$19,208,394	$803,740,032
Tajikistan	$436,373,874	$367,451,171	$10,120,562	$449,773,206
Tanzania	$2,701,211,659	$2,456,724,397	$50,005,557	$2,903,091,232
Togo	$310,160,274	$285,340,906	$5,611,456	$334,768,272
Tunisia	$981,411,231	$796,503,718	$21,582,152	$986,577,100
Turkey	$30,665,784,938	$23,659,504,785	$568,757,486	$28,931,632,252
Uganda	$1,674,499,634	$1,588,879,797	$385,265,851	$1,849,941,504
West Bank and Gaza	$211,482,529	$195,636,416	$6,930,262	$233,328,972
Yemen, Rep.	$1,096,736,296	$938,682,001	$21,313,571	$1,141,026,798
Zambia	$607,538,596	$576,387,647	$12,457,162	$675,092,378
Zimbabwe	$643,379,107	$617,966,425	$257,236,447	$715,488,782

Table SR3: Cost per Capita by Country per Scenario (2019 US$)

Country	Scenario 1: No mitigation	Scenario 2: Contact reduction: high symptomatic cases/low general population	Scenario 3: Contact reduction: high symptomatic cases/high general population	Scenario 4: 30-day lockdown + low contact reduction general population
Afghanistan	$35.29	$31.69	$0.69	$37.66
Algeria	$156.37	$113.61	$2.58	$144.09
Angola	$45.14	$43.35	$1.04	$50.86
Argentina	$359.01	$272.68	$6.70	$337.16
Bangladesh	$51.99	$45.53	$0.90	$54.30
Benin	$44.23	$40.34	$0.78	$47.44
Bolivia	$62.64	$53.55	$1.08	$65.06
Botswana	$168.67	$134.47	$3.75	$163.93
Brazil	$254.68	$191.02	$3.84	$237.90
Burkina Faso	$39.25	$36.44	$0.77	$42.53
Burundi	$29.56	$27.25	$0.54	$32.19
Cabo Verde	$59.74	$51.79	$3.64	$63.29
Cambodia	$41.75	$34.85	$0.64	$42.52
Cameroon	$37.67	$35.08	$0.75	$41.36
Central African Republic	$32.00	$29.37	$0.73	$34.60
Chad	$34.72	$30.45	$0.68	$36.92
Colombia	$226.59	$170.71	$3.24	$212.16
Comoros	$38.92	$35.66	$1.35	$42.39
Congo, Dem. Rep.	$33.80	$30.99	$0.55	$36.49
Congo, Rep.	$43.30	$40.41	$1.15	$47.65
Costa Rica	$280.05	$208.46	$4.54	$261.18
Cote d'Ivoire	$39.35	$35.51	$0.61	$42.28
Dominican Republic	$223.37	$172.09	$3.66	$212.38
Ecuador	$177.91	$139.37	$2.79	$170.24
Egypt, Arab Rep.	$55.61	$46.51	$1.14	$57.61
El Salvador	$80.82	$67.64	$1.69	$82.62
Equatorial Guinea	$153.78	$136.72	$4.46	$160.93
Eswatini	$53.74	$49.31	$2.43	$59.24
Ethiopia	$59.55	$51.53	$0.98	$61.19
Gabon	$149.83	$126.68	$3.62	$151.45
Gambia, The	$43.49	$39.81	$1.04	$46.54
Ghana	$45.61	$41.05	$0.73	$48.84
Guatemala	$106.51	$86.00	$1.85	$104.09
Guinea	$45.40	$41.04	$0.86	$48.41
Guinea-Bissau	$40.51	$37.35	$0.99	$43.93
Haiti	$49.92	$43.34	$0.89	$51.95
Honduras	$52.28	$46.89	$1.12	$55.70
India	$81.04	$70.12	$1.56	$84.06
Iraq	$134.87	$112.54	$2.34	$134.73
Jordan	$93.13	$75.06	$2.06	$91.30
Kenya	$67.80	$65.63	$1.57	$74.51
Lebanon	$217.12	$170.83	$4.85	$207.94
Lesotho	$41.20	$35.07	$0.87	$42.60
Liberia	$39.76	$36.12	$0.85	$42.59
Libya	$178.77	$137.16	$3.61	$170.74
Madagascar	$39.89	$35.39	$0.66	$42.20

Country	Scenario 1: No mitigation	Scenario 2: Contact reduction: high symptomatic cases/low general population	Scenario 3: Contact reduction: high symptomatic cases/high general population	Scenario 4: 30-day lockdown + low contact reduction general population
Malawi	$33.50	$30.27	$0.59	$35.92
Mali	$37.25	$32.63	$0.71	$39.60
Mauritania	$39.00	$35.86	$0.98	$42.28
Mauritius	$398.84	$293.12	$7.99	$367.56
Mexico	$250.48	$195.15	$4.38	$239.45
Morocco	$79.80	$67.48	$1.60	$81.61
Mozambique	$35.43	$31.90	$0.61	$37.95
Namibia	$94.03	$79.92	$2.21	$95.34
Nepal	$63.88	$54.18	$0.93	$65.05
Nicaragua	$50.65	$44.13	$1.02	$52.93
Niger	$30.40	$26.71	$0.57	$32.35
Nigeria	$43.52	$40.65	$0.83	$47.61
Pakistan	$37.28	$32.37	$0.72	$38.84
Paraguay	$158.96	$122.32	$2.42	$151.38
Peru	$207.17	$155.85	$2.81	$193.43
Rwanda	$42.46	$38.32	$0.81	$45.36
Sao Tome and Principe	$40.56	$41.08	$4.09	$47.59
Senegal	$40.86	$37.55	$0.85	$44.10
Sierra Leone	$38.44	$35.06	$0.76	$41.31
South Africa	$170.40	$144.25	$3.82	$174.88
Sri Lanka	$246.98	$187.59	$4.38	$230.21
Sudan	$38.81	$35.05	$0.70	$41.44
Syrian Arab Republic	$42.88	$40.43	$1.14	$47.54
Tajikistan	$47.95	$40.38	$1.11	$49.42
Tanzania	$47.96	$43.62	$0.89	$51.55
Togo	$39.32	$36.17	$0.71	$42.43
Tunisia	$84.86	$68.87	$1.87	$85.31
Turkey	$372.52	$287.41	$6.91	$351.45
Uganda	$39.19	$37.19	$9.02	$43.30
West Bank and Gaza	$46.29	$42.82	$1.52	$51.07
Yemen, Rep.	$38.48	$32.94	$0.75	$40.04
Zambia	$35.01	$33.22	$0.72	$38.91
Zimbabwe	$44.56	$42.80	$17.82	$49.55

Table SR4: Health System Costs of COVID-19 Response per Capita as % of GDP per Capita (Nominal)

Country	Scenario 1: No mitigation	Scenario 2: Contact reduction: high symptomatic cases/low general population	Scenario 3: Contact reduction: high symptomatic cases/high general population	Scenario 4: 30-day lockdown + low contact reduction general population
Afghanistan	6.77%	6.08%	0.13%	7.23%
Algeria	3.80%	2.76%	0.06%	3.50%
Angola	1.32%	1.26%	0.03%	1.48%
Argentina	3.07%	2.33%	0.06%	2.89%
Bangladesh	3.06%	2.68%	0.05%	3.20%
Benin	4.91%	4.47%	0.09%	5.26%
Bolivia	1.77%	1.51%	0.03%	1.83%
Botswana	2.04%	1.63%	0.05%	1.98%
Brazil	2.85%	2.14%	0.04%	2.67%
Burkina Faso	5.49%	5.10%	0.11%	5.95%
Burundi	10.88%	10.03%	0.20%	11.85%
Cabo Verde	1.64%	1.42%	0.10%	1.74%
Cambodia	2.76%	2.31%	0.04%	2.82%
Cameroon	2.46%	2.29%	0.05%	2.70%
Central African Republic	6.73%	6.17%	0.15%	7.27%
Chad	4.77%	4.18%	0.09%	5.07%
Colombia	3.40%	2.56%	0.05%	3.18%
Comoros	2.75%	2.52%	0.10%	3.00%
Congo, Dem. Rep.	6.02%	5.52%	0.10%	6.50%
Congo, Rep.	2.02%	1.88%	0.05%	2.22%
Costa Rica	2.33%	1.73%	0.04%	2.17%
Cote d'Ivoire	2.29%	2.07%	0.04%	2.46%
Dominican Republic	2.77%	2.14%	0.05%	2.64%
Ecuador	2.80%	2.20%	0.04%	2.68%
Egypt, Arab Rep.	2.18%	1.82%	0.04%	2.26%
El Salvador	1.99%	1.67%	0.04%	2.04%
Equatorial Guinea	1.50%	1.33%	0.04%	1.57%
Eswatini	1.30%	1.19%	0.06%	1.43%
Ethiopia	7.71%	6.67%	0.13%	7.92%
Gabon	1.88%	1.59%	0.05%	1.90%
Gambia, The	6.07%	5.56%	0.15%	6.50%
Ghana	2.07%	1.86%	0.03%	2.22%
Guatemala	2.34%	1.89%	0.04%	2.29%
Guinea	5.17%	4.67%	0.10%	5.51%
Guinea-Bissau	5.21%	4.80%	0.13%	5.65%
Haiti	5.75%	4.99%	0.10%	5.98%
Honduras	2.09%	1.88%	0.04%	2.23%
India	4.03%	3.49%	0.08%	4.18%
Jordan	2.20%	1.77%	0.05%	2.15%
Kenya	3.96%	3.84%	0.09%	4.36%
Lebanon	2.63%	2.07%	0.06%	2.51%
Lesotho	3.17%	2.70%	0.07%	3.28%
Liberia	5.87%	5.33%	0.13%	6.29%
Libya	2.47%	1.89%	0.05%	2.36%

Country	Scenario 1: No mitigation	Scenario 2: Contact reduction: high symptomatic cases/low general population	Scenario 3: Contact reduction: high symptomatic cases/high general population	Scenario 4: 30-day lockdown + low contact reduction general population
Madagascar	7.56%	6.71%	0.13%	8.00%
Malawi	8.60%	7.77%	0.15%	9.23%
Mali	4.14%	3.63%	0.08%	4.40%
Mauritania	3.28%	3.02%	0.08%	3.56%
Mauritius	3.55%	2.61%	0.07%	3.27%
Mexico	2.59%	2.02%	0.05%	2.48%
Morocco	2.46%	2.08%	0.05%	2.52%
Mozambique	7.10%	6.39%	0.12%	7.61%
Namibia	1.59%	1.35%	0.04%	1.61%
Nepal	6.18%	5.24%	0.09%	6.29%
Nicaragua	2.50%	2.18%	0.05%	2.61%
Niger	7.34%	6.45%	0.14%	7.81%
Nigeria	2.15%	2.00%	0.04%	2.35%
Pakistan	2.51%	2.18%	0.05%	2.62%
Paraguay	2.73%	2.10%	0.04%	2.60%
Peru	2.98%	2.25%	0.04%	2.79%
Rwanda	5.49%	4.96%	0.11%	5.87%
Sao Tome and Principe	2.03%	2.05%	0.20%	2.38%
Senegal	2.68%	2.47%	0.06%	2.90%
Sierra Leone	7.20%	6.57%	0.14%	7.74%
South Africa	2.67%	2.26%	0.06%	2.74%
Sri Lanka	6.02%	4.57%	0.11%	5.61%
Sudan	3.97%	3.59%	0.07%	4.24%
Syrian Arab Republic	2.11%	1.99%	0.06%	2.34%
Tajikistan	5.80%	4.88%	0.13%	5.98%
Tanzania	4.56%	4.15%	0.08%	4.91%
Togo	5.79%	5.32%	0.10%	6.25%
Tunisia	2.46%	2.00%	0.05%	2.47%
Turkey	3.98%	3.07%	0.07%	3.75%
Yemen, Rep.	4.07%	3.49%	0.08%	4.24%
Zambia	2.27%	2.16%	0.05%	2.53%
Zimbabwe	2.08%	1.99%	0.83%	2.31%

Table SR5: Health System Costs of COVID-19 Response per Capita as % of Total Health Spending per Capita (excluding out-of-pocket payments)

Country	Scenario 1: No mitigation	Scenario 2: Contact reduction: high symptomatic cases/low general population	Scenario 3: Contact reduction: high symptomatic cases/high general population	Scenario 4: 30-day lockdown + low contact reduction general population
Afghanistan	61.64%	55.35%	1.20%	65.78%
Algeria	60.05%	43.63%	0.99%	55.33%
Angola	47.41%	45.53%	1.09%	53.42%
Argentina	37.58%	28.55%	0.70%	35.30%
Bangladesh	151.94%	133.06%	2.63%	158.70%
Benin	145.48%	132.70%	2.58%	156.03%
Bolivia	29.41%	25.14%	0.51%	30.54%
Botswana	44.40%	35.39%	0.99%	43.15%
Brazil	25.07%	18.80%	0.38%	23.42%
Burkina Faso	95.87%	89.01%	1.88%	103.89%
Burundi	160.14%	147.61%	2.92%	174.36%
Cabo Verde	37.56%	32.56%	2.29%	39.79%
Cambodia	53.75%	44.86%	0.83%	54.75%
Cameroon	58.44%	54.41%	1.17%	64.16%
Central African Republic	195.59%	179.50%	4.44%	211.46%
Chad	109.56%	96.10%	2.13%	116.51%
Colombia	66.57%	50.15%	0.95%	62.33%
Comoros	65.97%	60.43%	2.28%	71.85%
Congo, Dem. Rep.	164.72%	151.06%	2.68%	177.84%
Congo, Rep.	61.53%	57.42%	1.64%	67.71%
Costa Rica	31.51%	23.45%	0.51%	29.38%
Cote d'Ivoire	58.24%	52.55%	0.90%	62.58%
Dominican Republic	53.93%	41.55%	0.88%	51.28%
Ecuador	35.25%	27.61%	0.55%	33.73%
Egypt, Arab Rep.	42.46%	35.51%	0.87%	43.99%
El Salvador	27.50%	23.02%	0.57%	28.11%
Equatorial Guinea	54.65%	48.59%	1.58%	57.19%
Eswatini	24.36%	22.36%	1.10%	26.86%
Ethiopia	216.36%	187.20%	3.56%	222.32%
Gabon	67.99%	57.49%	1.64%	68.73%
Gambia, The	207.80%	190.19%	4.97%	222.34%
Ghana	67.56%	60.80%	1.09%	72.35%
Guatemala	44.13%	35.63%	0.77%	43.13%
Guinea	121.20%	109.57%	2.29%	129.23%
Guinea-Bissau	103.72%	95.65%	2.53%	112.48%
Haiti	132.34%	114.90%	2.35%	137.71%
Honduras	26.20%	23.50%	0.56%	27.91%
India	129.22%	111.81%	2.48%	134.03%
Jordan	41.66%	33.58%	0.92%	40.84%
Kenya	102.40%	99.13%	2.37%	112.54%
Lebanon	32.79%	25.80%	0.73%	31.41%
Lesotho	48.17%	41.01%	1.02%	49.82%
Liberia	58.20%	52.88%	1.25%	62.34%

Country	Scenario 1: No mitigation	Scenario 2: Contact reduction: high symptomatic cases/low general population	Scenario 3: Contact reduction: high symptomatic cases/high general population	Scenario 4: 30-day lockdown + low contact reduction general population
Libya	57.19%	43.88%	1.15%	54.62%
Madagascar	165.38%	146.73%	2.74%	174.98%
Malawi	113.24%	102.30%	2.00%	121.42%
Mali	125.04%	109.54%	2.38%	132.94%
Mauritania	83.39%	76.67%	2.09%	90.39%
Mauritius	72.11%	53.00%	1.45%	66.46%
Mexico	54.24%	42.26%	0.95%	51.85%
Morocco	46.55%	39.36%	0.93%	47.60%
Mozambique	184.44%	166.07%	3.18%	197.59%
Namibia	23.35%	19.84%	0.55%	23.67%
Nepal	140.55%	119.20%	2.05%	143.13%
Nicaragua	26.92%	23.45%	0.54%	28.13%
Niger	134.00%	117.73%	2.51%	142.60%
Nigeria	54.85%	51.23%	1.05%	60.01%
Pakistan	94.19%	81.78%	1.82%	98.13%
Paraguay	48.58%	37.38%	0.74%	46.26%
Peru	65.47%	49.25%	0.89%	61.13%
Rwanda	88.32%	79.70%	1.69%	94.35%
Sao Tome and Principe	38.58%	39.07%	3.89%	45.26%
Senegal	77.67%	71.37%	1.61%	83.83%
Sierra Leone	44.54%	40.62%	0.88%	47.87%
South Africa	39.80%	33.69%	0.89%	40.84%
Sri Lanka	161.33%	122.53%	2.86%	150.37%
Sudan	25.53%	23.06%	0.46%	27.26%
Syrian Arab Republic	64.83%	61.12%	1.72%	71.88%
Tajikistan	86.09%	72.49%	2.00%	88.73%
Tanzania	135.11%	122.88%	2.50%	145.21%
Togo	101.40%	93.29%	1.83%	109.45%
Tunisia	33.08%	26.85%	0.73%	33.26%
Turkey	79.49%	61.33%	1.47%	74.99%
Yemen, Rep.	53.42%	45.72%	1.04%	55.58%
Zambia	61.92%	58.75%	1.27%	68.81%
Zimbabwe	47.43%	45.56%	18.96%	52.75%

Table SR6: Health System Costs of COVID-19 Response per Capita as % of Total Health Spending per Capita (including out-of-pocket payments)

Country	Scenario 1: No mitigation	Scenario 2: Contact reduction: high symptomatic cases/low general population	Scenario 3: Contact reduction: high symptomatic cases/high general population	Scenario 4: 30-day lockdown + low contact reduction general population
Afghanistan	34.75%	31.20%	0.68%	37.08%
Algeria	45.88%	33.33%	0.76%	42.28%
Angola	35.06%	33.67%	0.81%	39.51%
Argentina	32.46%	24.65%	0.61%	30.48%
Bangladesh	88.39%	77.41%	1.53%	92.33%
Benin	101.40%	92.48%	1.80%	108.75%
Bolivia	22.97%	19.64%	0.40%	23.86%
Botswana	42.18%	33.63%	0.94%	41.00%
Brazil	17.46%	13.10%	0.26%	16.31%
Burkina Faso	72.96%	67.74%	1.43%	79.07%
Burundi	122.70%	113.10%	2.24%	133.59%
Cabo Verde	29.80%	25.84%	1.81%	31.57%
Cambodia	33.90%	28.30%	0.52%	34.53%
Cameroon	34.47%	32.10%	0.69%	37.85%
Central African Republic	136.70%	125.45%	3.10%	147.79%
Chad	67.99%	59.63%	1.32%	72.30%
Colombia	55.40%	41.74%	0.79%	51.88%
Comoros	38.10%	34.91%	1.32%	41.50%
Congo, Dem. Rep.	119.85%	109.92%	1.95%	129.40%
Congo, Rep.	41.10%	38.35%	1.09%	45.23%
Costa Rica	25.79%	19.20%	0.42%	24.06%
Cote d'Ivoire	41.56%	37.50%	0.64%	44.65%
Dominican Republic	37.29%	28.73%	0.61%	35.46%
Ecuador	25.09%	19.65%	0.39%	24.01%
Egypt, Arab Rep.	26.21%	21.92%	0.54%	27.15%
El Salvador	21.63%	18.10%	0.45%	22.11%
Equatorial Guinea	31.62%	28.11%	0.92%	33.09%
Eswatini	22.17%	20.34%	1.00%	24.44%
Ethiopia	157.44%	136.22%	2.59%	161.78%
Gabon	55.50%	46.92%	1.34%	56.10%
Gambia, The	168.13%	153.88%	4.02%	179.90%
Ghana	49.02%	44.12%	0.79%	52.49%
Guatemala	28.78%	23.24%	0.50%	28.13%
Guinea	80.93%	73.16%	1.53%	86.29%
Guinea-Bissau	76.60%	70.64%	1.87%	83.07%
Haiti	93.38%	81.07%	1.66%	97.17%
Honduras	18.07%	16.21%	0.39%	19.25%
India	78.52%	67.94%	1.51%	81.44%
Jordan	32.55%	26.24%	0.72%	31.91%
Kenya	80.18%	77.62%	1.86%	88.12%
Lebanon	24.82%	19.52%	0.55%	23.77%
Lesotho	40.52%	34.50%	0.86%	41.90%
Liberia	39.52%	35.91%	0.85%	42.33%
Libya	57.19%	43.88%	1.15%	54.62%

Country	Scenario 1: No mitigation	Scenario 2: Contact reduction: high symptomatic cases/low general population	Scenario 3: Contact reduction: high symptomatic cases/high general population	Scenario 4: 30-day lockdown + low contact reduction general population
Madagascar	135.16%	119.91%	2.24%	143.01%
Malawi	101.66%	91.85%	1.80%	109.00%
Mali	92.43%	80.98%	1.76%	98.27%
Mauritania	55.26%	50.81%	1.39%	59.90%
Mauritius	48.67%	35.77%	0.98%	44.85%
Mexico	38.64%	30.10%	0.68%	36.94%
Morocco	31.32%	26.48%	0.63%	32.03%
Mozambique	171.29%	154.24%	2.95%	183.51%
Namibia	21.67%	18.42%	0.51%	21.97%
Nepal	90.42%	76.69%	1.32%	92.08%
Nicaragua	20.36%	17.74%	0.41%	21.27%
Niger	84.54%	74.27%	1.58%	89.96%
Nigeria	31.30%	29.24%	0.60%	34.25%
Pakistan	57.01%	49.49%	1.10%	59.39%
Paraguay	35.24%	27.12%	0.54%	33.56%
Peru	51.03%	38.39%	0.69%	47.65%
Rwanda	83.02%	74.92%	1.59%	88.69%
Sao Tome and Principe	33.72%	34.16%	3.40%	39.57%
Senegal	51.18%	47.02%	1.06%	55.23%
Sierra Leone	31.46%	28.70%	0.62%	33.82%
South Africa	36.93%	31.27%	0.83%	37.90%
Sri Lanka	107.46%	81.62%	1.91%	100.16%
Sudan	14.68%	13.26%	0.26%	15.68%
Syrian Arab Republic	64.83%	61.12%	1.72%	71.88%
Tajikistan	51.84%	43.65%	1.20%	53.43%
Tanzania	110.84%	100.81%	2.05%	119.13%
Togo	67.41%	62.02%	1.22%	72.76%
Tunisia	23.65%	19.19%	0.52%	23.77%
Turkey	68.25%	52.66%	1.27%	64.39%
Yemen, Rep.	29.52%	25.27%	0.57%	30.71%
Zambia	55.23%	52.40%	1.13%	61.37%
Zimbabwe	39.12%	37.58%	15.64%	43.51%

Table SR7: Health System Costs of COVID-19 Response per Capita as % of Government Health Spending per Capita

Country	Scenario 1: No mitigation	Scenario 2: Contact reduction: high symptomatic cases/low general population	Scenario 3: Contact reduction: high symptomatic cases/high general population	Scenario 4: 30-day lockdown + low contact reduction general population
Afghanistan	1202.08%	1079.37%	23.45%	1282.69%
Algeria	88.71%	81.75%	1.46%	81.75%
Angola	107.40%	121.02%	2.48%	121.02%
Argentina	50.50%	47.42%	0.94%	47.42%
Bangladesh	846.15%	883.78%	14.64%	883.78%
Benin	708.39%	759.74%	12.56%	759.74%
Bolivia	44.76%	46.49%	0.77%	46.49%
Botswana	79.37%	77.14%	1.76%	77.14%
Brazil	75.45%	70.48%	1.14%	70.48%
Burkina Faso	239.09%	259.10%	4.70%	259.10%
Burundi	549.78%	598.61%	10.02%	598.61%
Cabo Verde	66.14%	70.07%	4.03%	70.07%
Cambodia	246.48%	251.05%	3.79%	251.05%
Cameroon	438.21%	481.16%	8.76%	481.16%
Central African Republic	1316.05%	1422.83%	29.85%	1422.83%
Chad	580.66%	617.48%	11.31%	617.48%
Colombia	104.98%	98.29%	1.50%	98.29%
Comoros	453.20%	493.60%	15.69%	493.60%
Congo, Dem. Rep.	1344.28%	1451.34%	21.84%	1451.34%
Congo, Rep.	145.54%	160.17%	3.87%	160.17%
Costa Rica	42.14%	39.31%	0.68%	39.31%
Cote d'Ivoire	225.98%	242.80%	3.48%	242.80%
Dominican Republic	117.95%	112.14%	1.93%	112.14%
Ecuador	68.85%	65.88%	1.08%	65.88%
Egypt, Arab Rep.	144.91%	150.12%	2.96%	150.12%
El Salvador	42.66%	43.61%	0.89%	43.61%
Equatorial Guinea	232.43%	243.24%	6.74%	243.24%
Eswatini	35.15%	38.75%	1.59%	38.75%
Ethiopia	783.45%	805.02%	12.88%	805.02%
Gabon	105.27%	106.41%	2.54%	106.41%
Gambia, The	1119.18%	1197.51%	26.78%	1197.51%
Ghana	176.13%	188.62%	2.84%	188.62%
Guatemala	118.58%	115.89%	2.06%	115.89%
Guinea	985.52%	1050.76%	18.65%	1050.76%
Guinea-Bissau	234.39%	254.19%	5.71%	254.19%
Haiti	863.00%	898.02%	15.33%	898.02%
Honduras	57.07%	60.80%	1.23%	60.80%
India	508.22%	527.12%	9.75%	527.12%
Jordan	65.90%	64.60%	1.46%	64.60%
Kenya	283.05%	311.08%	6.56%	311.08%
Lebanon	62.90%	60.24%	1.40%	60.24%
Lesotho	75.50%	78.08%	1.59%	78.08%
Liberia	408.55%	437.58%	8.76%	437.58%

Country	Scenario 1: No mitigation	Scenario 2: Contact reduction: high symptomatic cases/low general population	Scenario 3: Contact reduction: high symptomatic cases/high general population	Scenario 4: 30-day lockdown + low contact reduction general population
Libya	90.36%	86.30%	1.82%	86.30%
Madagascar	347.06%	367.20%	5.74%	367.20%
Malawi	403.73%	432.89%	7.13%	432.89%
Mali	395.72%	420.70%	7.52%	420.70%
Mauritania	227.51%	246.63%	5.71%	246.63%
Mauritius	163.47%	150.65%	3.28%	150.65%
Mexico	104.02%	99.44%	1.82%	99.44%
Morocco	99.34%	101.59%	1.99%	101.59%
Mozambique	345.76%	370.42%	5.96%	370.42%
Namibia	37.72%	38.24%	0.89%	38.24%
Nepal	756.48%	770.34%	11.03%	770.34%
Nicaragua	43.88%	45.85%	0.88%	45.85%
Niger	551.71%	587.10%	10.34%	587.10%
Nigeria	421.11%	460.77%	8.08%	460.77%
Pakistan	337.64%	351.76%	6.51%	351.76%
Paraguay	94.10%	89.61%	1.43%	89.61%
Peru	102.18%	95.40%	1.39%	95.40%
Rwanda	260.66%	278.46%	4.99%	278.46%
Sao Tome and Principe	96.69%	113.46%	9.75%	113.46%
Senegal	224.71%	242.51%	4.65%	242.51%
Sierra Leone	398.63%	428.42%	7.84%	428.42%
South Africa	74.07%	76.01%	1.66%	76.01%
Sri Lanka	374.37%	348.94%	6.64%	348.94%
Sudan	131.01%	139.90%	2.36%	139.90%
Syrian Arab Republic	143.17%	158.74%	3.79%	158.74%
Tajikistan	301.02%	310.26%	6.98%	310.26%
Tanzania	332.58%	357.44%	6.16%	357.44%
Togo	505.79%	545.92%	9.15%	545.92%
Tunisia	58.41%	58.72%	1.28%	58.72%
Turkey	101.33%	95.60%	1.88%	95.60%
Yemen, Rep.	524.63%	545.82%	10.20%	545.82%
Zambia	161.72%	179.70%	3.32%	179.70%
Zimbabwe	101.99%	113.43%	40.78%	113.43%

Table SR8. Average Cost per Capita by Country Income Category (2019 US$): Sensitivity Analysis on % of Symptomatic Cases Tested

	Scenario 1: No mitigation	Scenario 2: Contact reduction: high symptomatic cases/low general population	Scenario 3: Contact reduction: high symptomatic cases/high general population	Scenario 4: 30-day lockdown + low contact reduction general population
Average Cost per Capita by Country Income Category (2019 US$): 20% of Symptomatic Cases Tested				
Low Income Countries (LIC)	$43.19	$45.54	$1.55	$53.93
Lower-Middle Income Countries (LMIC)	$52.63	$53.89	$1.27	$64.25
Upper-Middle Income Countries (UMIC)	$75.57	$63.65	$1.39	$78.21
Average Cost per Capita by Country Income Category (2019 US$): 40% of Symptomatic Cases Tested				
Low Income Countries (LIC)	$43.19	$59.57	$2.01	$70.32
Lower-Middle Income Countries (LMIC)	$52.63	$69.75	$1.61	$82.78
Upper-Middle Income Countries (UMIC)	$75.57	$74.79	$1.60	$91.38
Average Cost per Capita by Country Income Category (2019 US$): 60% of Symptomatic Cases Tested				
Low Income Countries (LIC)	$43.19	$73.60	$2.46	$86.72
Lower-Middle Income Countries (LMIC)	$52.63	$85.61	$1.95	$101.32
Upper-Middle Income Countries (UMIC)	$75.57	$85.93	$1.81	$104.55
Average Cost per Capita by Country Income Category (2019 US$): 80% of Symptomatic Cases Tested				
Low Income Countries (LIC)	$43.19	$87.63	$2.92	$103.11
Lower-Middle Income Countries (LMIC)	$52.63	$101.48	$2.29	$119.85
Upper-Middle Income Countries (UMIC)	$75.57	$97.08	$2.02	$117.72
Average Cost per Capita by Country Income Category (2019 US$): 100% of Symptomatic Cases Tested				
Low Income Countries (LIC)	$43.19	$101.66	$3.38	$119.50
Lower-Middle Income Countries (LMIC)	$52.63	$117.34	$2.62	$138.38
Upper-Middle Income Countries (UMIC)	$75.57	$108.22	$2.22	$130.89
